# Unexpected enrichment of antibiotic resistance genes in recirculating aquaculture effluents of rainbow trout (*Oncorhynchus mykiss*): microbial community assembly and mobile genetic elements as key drivers

**DOI:** 10.3389/fmicb.2026.1913067

**Published:** 2026-08-12

**Authors:** Xiaofei Yang, Lili Li, Jinxin Zhang, Jiangqi Qu, Shaogang Xu

**Affiliations:** 1 National Engineering Research Center for Freshwaters, Fisheries Science Institute, Beijing Academy of Agricultural and Forestry Sciences, Beijing, China; 2 National Key Laboratory of Intelligent Tracking and Forecasting for Infectious Diseases (NITFID), NHC Key Laboratory for Medical Virology and Viral Diseases, National Institute for Viral Disease Control and Prevention, Chinese Center for Disease Control and Prevention, Beijing, China

**Keywords:** antibiotic resistance genes (ARGs), aquaculture effluent, microbial community, mobile genetic elements (MGEs), rainbow trout (*oncorhynchus mykiss*), recirculating aquaculture system

## Abstract

**Introduction:**

Recirculating aquaculture systems (RAS) are increasingly used in intensive aquaculture because of their capacity to reduce nitrogen, phosphorus, and organic matter discharges. However, whether improved conventional effluent quality is accompanied by a reduced antibiotic resistance gene (ARG) burden remains unclear.

**Methods:**

During the mid-cultivation stage, effluents were collected from four RAS farms and four paved flow-through raceway systems (PFRS). Water quality parameters, including total nitrogen (TN), total phosphorus (TP), chemical oxygen demand (COD), chlorophyll-a (Chl-a), and dissolved oxygen (DO), were measured. Microbial community composition was characterized using 16S rRNA gene sequencing, while ARGs and mobile genetic elements (MGEs) were quantified using GeoChip 5.0. Multivariate statistics and co-occurrence network analyses were used to evaluate associations among environmental conditions, microbial communities, ARG profiles, and MGE profiles.

**Results:**

RAS effluents showed substantially lower nutrient and organic loads than PFRS, confirming improved conventional water quality. However, total ARG abundance was significantly higher in RAS than in PFRS, increasing from 35.39 ± 11.43 × 10^6^ to 63.99 ± 15.33 × 10^6^, corresponding to a 1.81-fold increase. In contrast, total MGE abundance was numerically higher in RAS but did not differ significantly between systems. Microbial alpha diversity was lower in RAS, and beta diversity analysis showed distinct community structuring between cultivation modes, with both compositional differences and dispersion effects contributing to group separation. ARG class- and subtype-level profiles showed heterogeneous abundance patterns, with several major ARG categories remaining highly abundant in RAS, although most feature-level differences were not significant after FDR correction. Co-occurrence network analysis further revealed structured associations among microbial species, ARGs, and MGEs, with modular organization and highly connected resistance-associated nodes.

**Conclusion:**

This study demonstrates a “low-pollution but non-reduced ARG burden” pattern in RAS effluents. Improved removal of conventional pollutants did not correspond to a proportional reduction in ARG or MGE profiles. These findings indicate that conventional water-quality indicators alone are insufficient to evaluate resistance-associated genetic pollution in engineered aquaculture systems, and that ARGs, MGEs, microbial community structure, and species–ARG/MGE association patterns should be integrated into future RAS monitoring and management.

## 1 Introduction

Aquaculture plays a pivotal role in global protein supply, food security, and human nutrition. Under increasing pressure from population growth and limited terrestrial resources, aquaculture production has progressively shifted toward intensive and engineering-driven systems (Khan et al., 2025). Traditional pond-based culture, although widely adopted due to open water exchange and frequent renewal, typically exhibits low water-use efficiency. Effluents from these systems often contain elevated concentrations of nitrogen, phosphorus, and organic matter, contributing to eutrophication, ecological imbalance, and the dissemination of pathogenic microorganisms in receiving water bodies (Kashem et al., 2023; Niu et al., 2025). Amid tightening environmental regulations and growing water resource constraints, the sustainability of such high-emission production modes has been increasingly questioned (Wang and Wang, 2022). Consequently, RAS have been rapidly adopted for high-value species production. By integrating mechanical filtration, biofiltration, and water recirculation units, RAS substantially reduce nutrient and organic discharges and represent a key technological pathway toward high-density and low-emission aquaculture (Taufik et al., 2024; Hashmi et al., 2025).

In parallel with improvements in water treatment technologies, ARGs have emerged as a novel class of environmental pollutants because of their persistence, mobility, and potential risks to ecosystems and public health (Klümper et al., 2025). Unlike conventional chemical contaminants, ARGs are replicable genetic elements that can spread within microbial communities via HGT, mediated by integrons, plasmids, and transposons, collectively referred to as MGEs (Liu et al., 2025; Jing et al., 2026). Under sustained anthropogenic pressures, particularly in aquatic environments receiving urban wastewater, agricultural runoff, and aquaculture effluents, water bodies have become important reservoirs and transmission pathways for antibiotic resistance (Popoola et al., 2025).

Aquaculture systems may contribute to the environmental dissemination of resistance-related genetic elements through multiple ecological and hydrological pathways. Resistance-associated bacteria and ARGs can occur across aquaculture water, sediments, biofilms, and animal-associated microbiota, reflecting the close coupling between production environments and microbial genetic pools (Basili et al., 2026). Importantly, improvements in physicochemical water quality do not necessarily imply reduced microbial or genetic risks. Aquaculture effluent functions as the interface between production systems and natural ecosystems and is not merely a passive carrier of resistance genes (Milijasevic et al., 2024). Instead, effluents may facilitate the release and redistribution of microbial cells and genetic material through hydraulic transport, particle export, biofilm detachment, and resuspension processes (Sooriyakumar et al., 2022). Under certain engineering and hydrological conditions, effluent discharge may therefore serve as a conduit for ARG dissemination into surrounding environments, highlighting the need to evaluate microbial and genetic indicators alongside conventional water-quality parameters (Grenni, 2022).

To date, research on RAS has primarily focused on conventional pollutant removal (Guo et al., 2026), whereas microbial ecological dynamics and genetic risk structures remain comparatively understudied. The operational characteristics of RAS, including extended hydraulic retention times, closed-loop circulation, and extensive biofilm development within biofilters, promote stable and densely structured microbial communities (Na et al., 2018). Biofilm microenvironments, characterized by high cell density, close cellular proximity, and abundant extracellular polymeric substances (EPS), may facilitate microbial persistence and create conditions favorable for potential gene exchange (Mirghani et al., 2022). Under such structured microbial habitats, integrons and other mobile genetic elements (MGEs) may be retained together with antibiotic resistance genes ARGs, contributing to ARG persistence even when direct antibiotic inputs are limited or not apparent (Wang et al., 2025). These observations raise the possibility that improved conventional water quality may not always correspond to reduced resistance-related genetic indicators in engineered aquaculture systems.

Among high-value cold-water species, rainbow trout (*Oncorhynchus mykiss*) is widely cultured because of its rapid growth and high nutritional quality, and has become an important species in Chinese aquaculture (Wang et al., 2026). In commercial production, PFRS and RAS are the two predominant culture modes for rainbow trout. PFRS exhibit high water exchange and open-system characteristics, with direct effluent discharge, whereas RAS rely on closed-loop circulation and engineered treatment units to reduce pollutants and optimize water use efficiency (Liu et al., 2021). These contrasting operational modes generate distinct hydraulic regimes, microbial community structures, and environmental selection pressures (Milián-Sorribes et al., 2021). Consequently, trout farm effluents may function not only as carriers of conventional pollutants but also as pathways for the release and redistribution of microbial cells and resistance-related genetic elements (Rossi et al., 2025). However, systematic comparisons of ARG and MGE profiles between commercial-scale RAS and PFRS effluents remain scarce. Existing studies have largely focused on antibiotic residues, cultivable resistant bacteria, or isolated resistance indicators, with limited integration of microbial community structure, ARG profiles, and MGE profiles at the effluent-system level (Manyi-Loh et al., 2018). In particular, empirical evidence remains limited regarding whether reductions in conventional pollutants are accompanied by corresponding decreases in resistance-related genetic indicators (La Rosa et al., 2025).

In this study, we conducted a comparative investigation of effluents from commercial rainbow trout farms operated under RAS and PFRS modes. We hypothesized that RAS would reduce conventional pollutant loads compared with PFRS, but that reduced pollutant concentrations would not necessarily correspond to lower ARG burden because recirculating conditions may alter microbial community structure and retain selected resistance-related genetic elements. By integrating physicochemical water-quality analysis, 16S rRNA gene sequencing, and GeoChip 5.0-based profiling of ARGs and MGEs, this study aimed to: (1) compare water-quality characteristics and ARG burden between RAS and PFRS effluents; (2) evaluate associations among environmental variables, microbial community structure, ARG profiles, and MGE profiles; and (3) explore ecological associations potentially underlying ARG persistence under low-pollution recirculating conditions, thereby providing a scientific basis for optimizing aquaculture system design and improving resistance-risk assessment.

## 2 Materials and methods

### 2.1 Sampling design and site selection

This study employed a cross-system comparative design to evaluate associations among environmental conditions, microbial community structure, MGE profiles, and ARG profiles under comparable cultivation-stage and management conditions. Rainbow trout (*Oncorhynchus mykiss*) was selected as the target species because it is a representative cold-water aquaculture species requiring relatively stable thermal conditions, high dissolved oxygen (DO), and controlled hydraulic environments for intensive production.

Commercial rainbow trout farms located in northern China were selected for investigation. Two major cultivation modes were compared: PFRS and RAS. Farms were selected based on predefined inclusion criteria, including stable commercial production, comparable stocking density and cultivation stage, absence of major disease outbreaks during the sampling period, absence of emergency treatment immediately before sampling, and availability of farm-management records.

**FIGURE 1:**
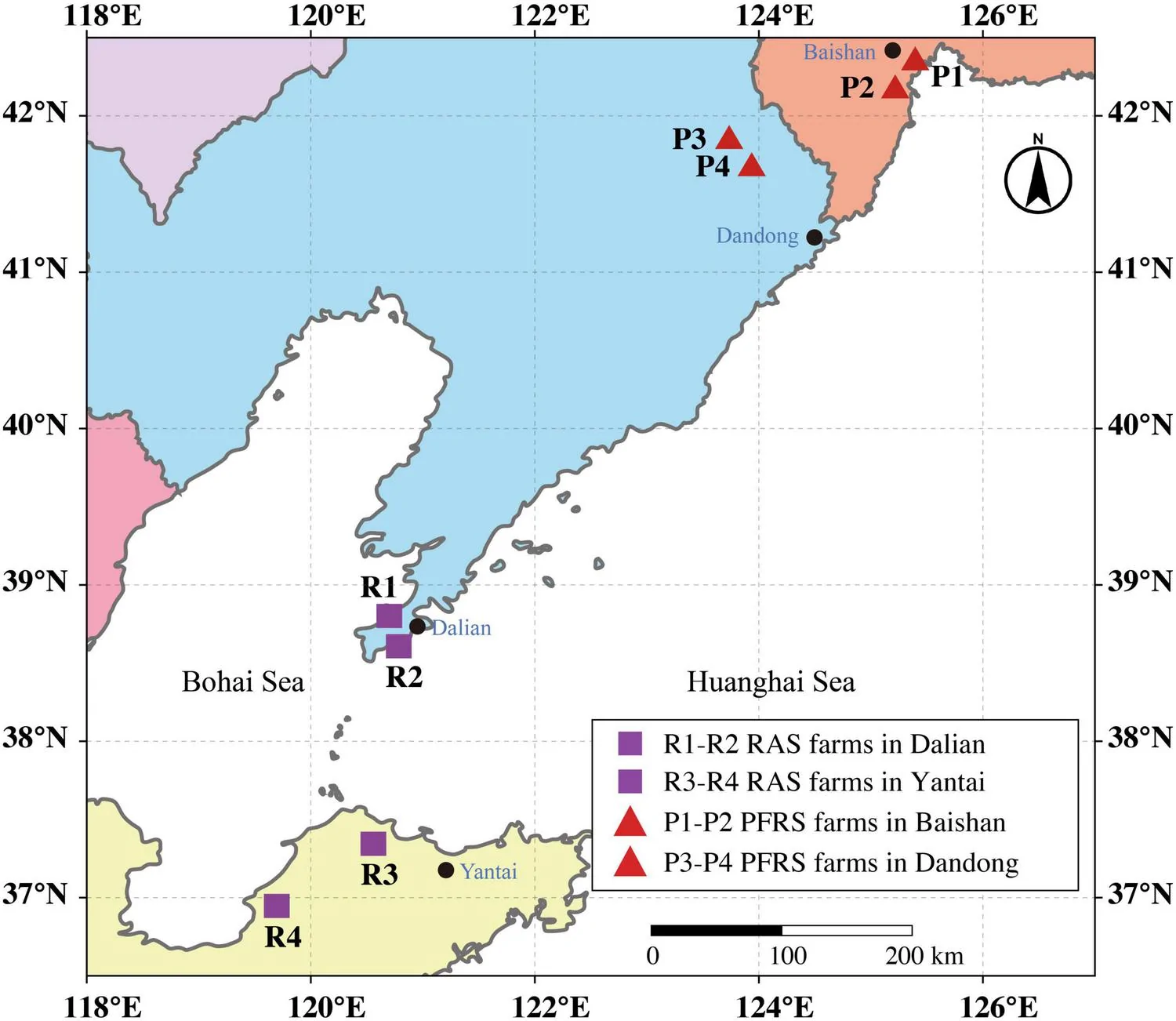
The geographic locations of sampling sites within the Bohai Sea and Yellow Sea regions of China.

A total of eight independent commercial farms were included, comprising four PFRS farms and four RAS farms (Figure 1). Each farm represented an independently managed production system and was treated as one biological replicate (*n* = 4 per system). Because commercial rainbow trout RAS farms remain relatively limited and geographically concentrated in northern China due to the high technical requirements of cold-water aquaculture and the relatively recent development of large-scale commercial RAS production, all farms meeting the predefined criteria during the sampling period were included. Therefore, this study adopted a full-coverage sampling strategy within the defined commercial production scope, thereby minimizing selection bias and maximizing representativeness. Sampling was conducted as a single cross-sectional survey during the mid-cultivation stage, when fish biomass, feeding conditions, and system operation were relatively stable. At each farm, three spatially separated effluent subsamples were collected from the main outlet and combined into one composite sample. These subsamples were used to reduce microscale spatial heterogeneity and were not treated as independent biological replicates.

Feed and medication information was obtained from farm managers and production records. During the documented pre-sampling period, all farms used commercial extruded feed formulated for rainbow trout. No medicated feed, antibiotic growth promoters, probiotic-containing feed additives, or immune-enhancing bioactive feed supplements were reported in the available farm records. In addition, no routine prophylactic antibiotic treatment was reported. These observations are consistent with current trends toward regulated and reduced antimicrobial use in aquaculture production; however, because antibiotic residues and feed components were not directly chemically analyzed, unrecorded historical exposure or low-level inputs cannot be completely excluded (Supplementary Table 1).

### 2.2 Sample collection

Sampling was conducted during the mid-cultivation stage, when average fish weight was approximately 500 g, feeding rates were stable, and major operational parameters had reached relatively consistent conditions across farms. This sampling stage was selected to minimize variation associated with early system establishment or harvest-related disturbance and to characterize microbial community and resistance-related genetic patterns under routine operational conditions.

At each farm, effluent samples were collected from the main discharge outlet representing the final water output of each production system. Three spatially separated subsamples were collected from the upper water layer (0–30 cm) and combined into one composite sample for subsequent analysis. These subsamples were considered technical subsamples for reducing microscale spatial heterogeneity and were not treated as independent biological replicates. The biological replicate unit was defined at the farm level, with each farm representing an independently managed aquaculture system. After collection, samples were immediately transported on ice to the laboratory and processed within 6 h. For microbial community and ARG/MGE analyses, water samples were filtered through sterile 0.22 μm membrane filters, and filters were stored at −80 °C until DNA extraction. Separate water aliquots were stored at 4 °C for physicochemical analyses within 24 h. Water temperature, dissolved oxygen (DO), pH, flow rate, stocking density, and feeding rate were recorded simultaneously to characterize operational conditions and construct the environmental variable matrix for subsequent multivariate analyses.

### 2.3 Water quality measurement and characterization of environmental selection pressure

To characterize environmental conditions associated with different aquaculture modes, key physicochemical parameters, including TN, TP, COD, and Chl-a, were measured. These parameters were selected to represent nutrient loading, organic matter accumulation, and trophic status of aquaculture effluents, which are important factors influencing microbial habitat conditions and community organization (Zhang et al., 2025).

TN, TP, COD, and Chl-a were determined according to standard analytical procedures described in Standard Methods for the Examination of Water and Wastewater (23rd Edition) (APHA et al., 2017). Temperature, DO, and pH were measured *in situ* using a calibrated multiparameter water-quality meter (YSI ProQuatro, YSI Incorporated, USA). Each laboratory-measured parameter was analyzed in triplicate, and the mean values were used as representative measurements for each farm. The resulting physicochemical dataset was used to compare environmental characteristics between RAS and PFRS and to evaluate associations between environmental variation and microbial community structure, ARG profiles, and MGE profiles through multivariate analyses. Because antibiotic residues, metals, disinfectants, and other potential co-selective agents were not directly measured, physicochemical parameters were interpreted as environmental characteristics rather than direct selective drivers of ARG enrichment.

### 2.4 Microbial community sequencing and ecological differentiation assessment

Microbial community structure was characterized by high-throughput sequencing of the V4 region of the bacterial 16S rRNA gene. Total genomic DNA was extracted from 0.22 μm membrane filters using the DNeasy PowerWater Kit (Qiagen, Germany). DNA concentration and purity were assessed using a NanoDrop 2000 spectrophotometer (Thermo Scientific, USA) and Qubit 4.0 fluorometer (Thermo Scientific, USA). The V4 region of the bacterial 16S rRNA gene was amplified using the 515F/806R primer pair following previously described protocols (Katiraei et al., 2022). Amplicons were purified using the AMPure XP system (Beckman Coulter, USA), quantified, pooled at equimolar concentrations, and sequenced on an Illumina NovaSeq 6000 platform with paired-end 250 bp reads.

Raw sequencing reads were quality-filtered, trimmed, and screened for chimeric sequences using standard bioinformatic pipelines. High-quality sequences were clustered into operational taxonomic units (OTUs) at 97% sequence similarity. Representative OTU sequences were taxonomically classified against the SILVA database (version 138) (Quast et al., 2013). Sequencing quality-control information, including raw reads, quality-filtered reads, effective sequences, OTU numbers, and sequencing coverage, is provided in Supplementary Table 2.

Alpha diversity indices, including observed OTUs, Chao1 richness, ACE richness, Shannon diversity, and Pielou’s evenness, were calculated using the phyloseq and vegan packages in R (McMurdie and Holmes, 2013). Beta diversity was evaluated using Bray–Curtis dissimilarity matrices based on OTU abundance profiles. Principal coordinate analysis (PCoA) was performed to visualize differences in microbial community composition between RAS and PFRS. Community differentiation was assessed using ANOSIM and PERMANOVA with 999 permutations, and homogeneity of multivariate dispersion was evaluated using PERMDISP before interpreting PERMANOVA results.

Differentially abundant taxa between RAS and PFRS were identified using linear discriminant analysis effect size (LEfSe) analysis (Segata et al., 2011). The top 30 most abundant taxa, ranked by relative abundance across all samples, were defined as dominant taxa and selected for subsequent taxon–ARG/MGE association analyses. Microbial functional potential and phenotypic traits were predicted from OTU abundance profiles using PICRUSt2 (version 2.5.0) and BugBase, respectively (Douglas et al., 2020).

### 2.5 ARGs and MGEs detection and resistance load assessment

ARGs and MGEs were profiled using the GeoChip 5.0 functional gene microarray (Mega Inc., China), which targets multiple functional gene categories associated with antibiotic resistance and mobile genetic elements, including integrons, transposons, insertion sequences, plasmid-associated markers, and other resistance-related determinants (Shi et al., 2019).

Sample DNA was fluorescently labeled, hybridized to the GeoChip 5.0 microarray, and scanned using an Axon GenePix 4300A scanner according to the standardized GeoChip analytical workflow. Raw signal intensities were subjected to background correction, quality filtering, and normalization. The processed hybridization signals were further converted into absolute abundance values following the quantitative calculation procedures provided by the GeoChip service platform. ARG and MGE abundance profiles were subsequently analyzed at multiple hierarchical levels, including individual subtypes, functional classes, and total abundance. These absolute abundance datasets were used for comparative analyses between RAS and PFRS systems and integrated with microbial community data for subsequent ecological association analyses.

### 2.6 Community–ARGs structural matching and network coupling analysis

To evaluate associations among microbial community structure, ARG profiles, and MGE profiles, a multi-level analytical framework was implemented. Mantel tests were performed to assess correlations between Bray–Curtis dissimilarity matrices derived from species-level microbial community composition and ARG/MGE abundance profiles. Procrustes analysis was conducted to evaluate the concordance between microbial community ordination patterns and ARG composition (Liu et al., 2024). Species–ARG and species–MGE associations were evaluated using Spearman rank correlation analysis. Multiple comparisons were corrected using the Benjamini–Hochberg false discovery rate (FDR) procedure, and FDR-adjusted q values were used to determine statistical significance (Niu et al., 2024). Co-occurrence networks were constructed based on significant Spearman correlations (| ρ| ≥ 0.7, FDR-adjusted *q* < 0.05) among microbial species, ARGs, and MGEs. Network nodes represented microbial species, ARGs, and MGEs, whereas network edges represented statistically significant species–ARG/MGE associations. Network topology parameters, including node degree, betweenness centrality, and modularity, were calculated to evaluate network organization (Lee et al., 2022). Network visualization and topology analysis were performed using Gephi (version 0.9.2), and network modules were identified using the Louvain community detection algorithm implemented in Gephi. Detailed network construction parameters, topology metrics, significant associations, and node centrality values are provided in Supplementary Table 5A–C.

### 2.7 Statistical analysis and causal inference considerations

All statistical analyses were performed using R software (version 4.3.1). Differences in physicochemical parameters, alpha diversity indices, ARG profiles, and MGE profiles between RAS and PFRS were evaluated using independent-sample statistical tests. Student’s *t*-test or Welch’s *t*-test was applied according to data distribution and variance homogeneity, whereas the Wilcoxon rank-sum test was used when parametric assumptions were not satisfied. Absolute abundance values of ARGs and MGEs were used directly for between-system comparisons. For predefined ecological indicators, including physicochemical parameters, total ARG abundance, total MGE abundance, and alpha diversity indices, conventional statistical tests were applied, and effect sizes with confidence intervals were calculated where applicable. For analyses involving multiple feature-level comparisons, including ARG classes, ARG/MGE subtypes, taxonomic associations, and species–ARG/MGE correlation analyses, P values were adjusted using the Benjamini–Hochberg false discovery rate (FDR) procedure, and FDR-adjusted q values were reported. Multivariate analyses were performed based on Bray–Curtis dissimilarity matrices. PERMANOVA was conducted with 999 permutations, and homogeneity of multivariate dispersion was evaluated using PERMDISP before interpreting PERMANOVA results. All statistical tests were two-sided, with significance thresholds set at *P* < 0.05 or FDR-adjusted *q* < 0.05 where applicable.

## 3 Results

### 3.1 Divergent environmental selection pressures in effluents driven by cultivation mode

**FIGURE 2:**
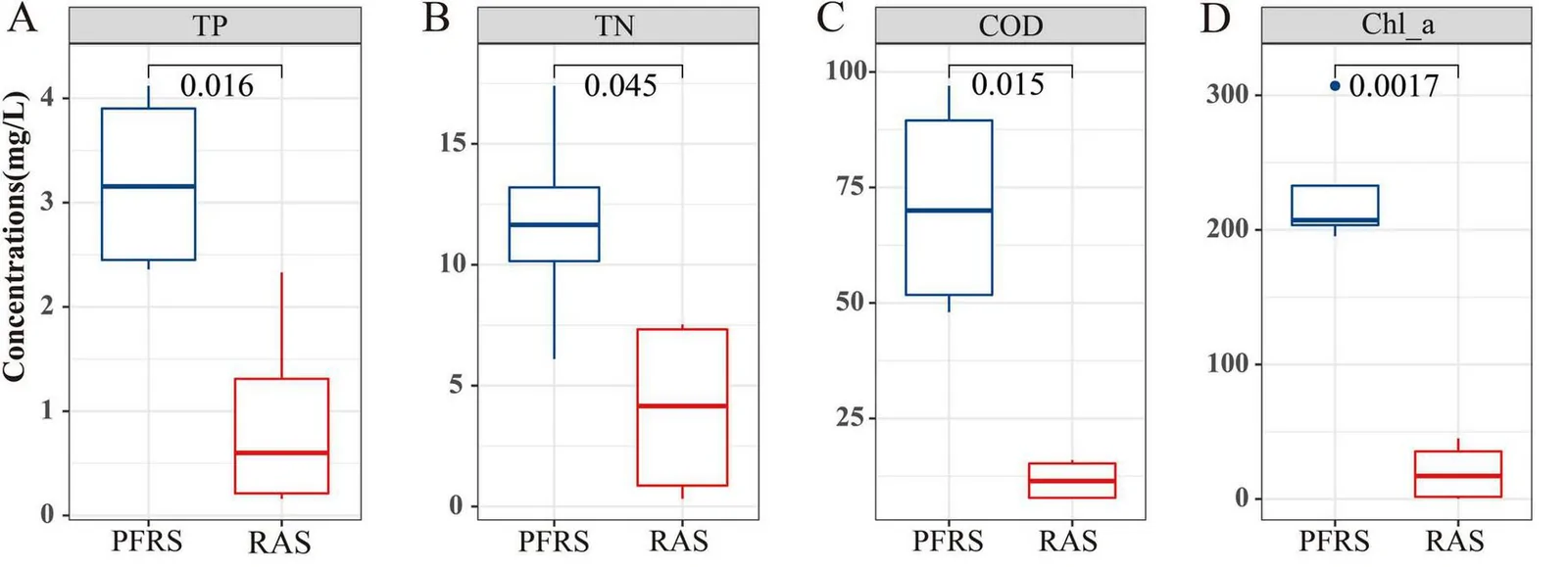
Comparison of key water quality parameters between the PFRS and RAS groups **(A–D)**. Box plots show the median (central line), 25th–75th percentiles (boxes), and 1.5 × interquartile range (whiskers). The *p*-values above each panel indicate the significance level from the independent samples *t*-test (*p* < 0.05 denotes a significant difference).

The two rainbow trout aquaculture systems generated markedly different environmental selection gradients at the effluent level, providing a clear ecological context for subsequent microbial community assembly and ARG dynamics. Effluents from the PFRS exhibited significantly higher nutrient and organic pollution loads than those from the RAS (*P* < 0.05) (Figure 2). Mean concentrations of TP, TN, and COD in PFRS were 3.20, 11.70, and 71.25 mg L^–1^, respectively, whereas corresponding values in RAS were 0.92, 4.04, and 11.63 mg L^–1^. The mean Chl-a concentration was 229.17 ug L^–1^ in PFRS and 19.87 ug L^–1^ in RAS (Figures 2A–C). Chl-a median values further emphasized system differentiation, reaching approximately 229.17 μg L^–1^ in PFRS compared with about 19.87 μg L^–1^ in RAS, indicating pronounced differences in autotrophic biomass and eutrophication status (Figure 2D). DO concentrations in RAS effluents (9.36 ± 1.49 mg L^–1^) were significantly higher than those in PFRS (8.63 ± 1.07 mg L^–1^), suggesting a more oxidized and environmentally stable water condition. Overall, the two systems differed not in a single water quality parameter but in an integrated environmental gradient shaped by multiple coordinated factors. PFRS effluents were characterized by high nutrient availability, elevated organic loading, relatively lower oxygen conditions, and stronger hydrological disturbance, whereas RAS effluents reflected low nutrient and organic loads, higher oxygen availability, and greater environmental stability associated with system closure. This composite gradient provides the primary ecological framework governing environmental selection, microbial community differentiation, and subsequent ARGs evolutionary dynamics.

### 3.2 Environmental filtering reshapes microbial community assembly

**FIGURE 3:**
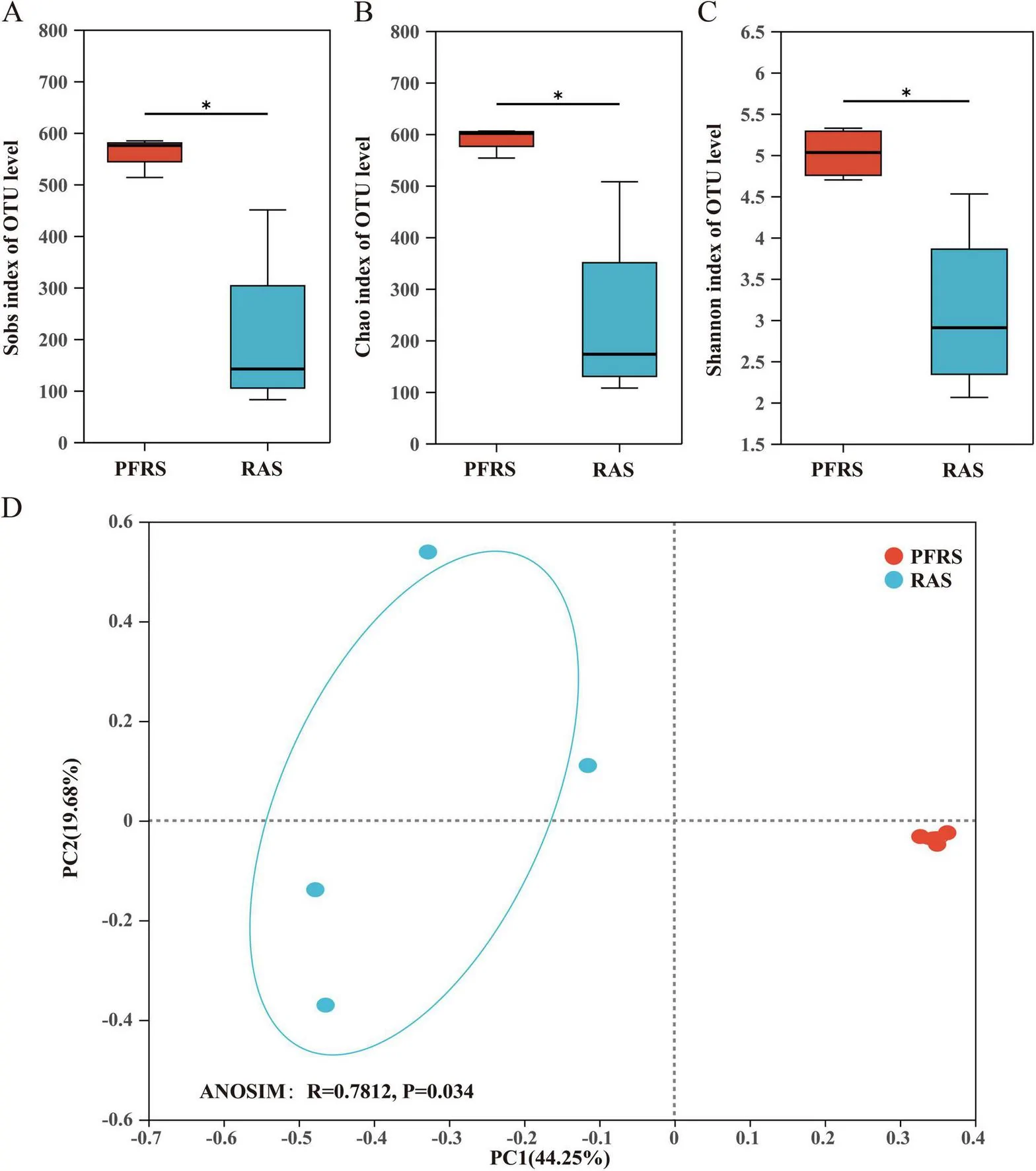
Comparison of bacterial diversity and community structure between the PFRS and RAS groups. Alpha diversity **(A–C)** and PCoA ordination **(D)** are shown. Significant differences are marked with *p < 0.05. ANOSIM confirmed significant community dissimilarity (*R* = 0.7812, *p* = 0.034).

Microbial diversity and community composition differed substantially between RAS and PFRS effluents, indicating distinct ecological conditions associated with different cultivation modes (Supplementary Tables 2A–C). Alpha diversity analysis showed consistently higher microbial richness and diversity in PFRS than in RAS. Sobs, Chao1 richness, Shannon diversity, and Pielou’s evenness were significantly higher in PFRS effluents than in RAS (Welch’s *t*-test, *P* < 0.05; Figures 3A–C). The corresponding effect sizes were large (Hedges’ g = −1.92 to −2.59), with bootstrap confidence intervals of RAS/PFRS fold ratios consistently below 1, indicating pronounced reductions in richness and evenness under RAS conditions. Beta diversity analysis further demonstrated distinct microbial community structures between the two cultivation modes. PCoA based on Bray–Curtis dissimilarity matrices showed clear separation of RAS and PFRS samples (Figure 3D). PERMANOVA confirmed significant differences in overall community composition between cultivation modes (pseudo-F = 4.287, R^2^ = 0.417, *P* = 0.028, 999 permutations), indicating that cultivation mode explained a substantial proportion of microbial community variation. However, PERMDISP analysis also revealed significant differences in within-group dispersion (*F* = 66.277, *P* = 0.028), suggesting that variation in community heterogeneity contributed to the observed PERMANOVA pattern. Therefore, the separation between RAS and PFRS communities reflects both differences in community composition and differences in within-system ecological variability rather than a simple shift in community centroid alone. Compared with PFRS, RAS exhibited reduced microbial richness and evenness, accompanied by a distinct, lower-diversity community structure with altered within-system dispersion under recirculating operation. These cultivation-associated differences provide an ecological foundation for subsequent analyses of ARG and MGE distribution patterns across contrasting aquaculture systems.

**FIGURE 4:**
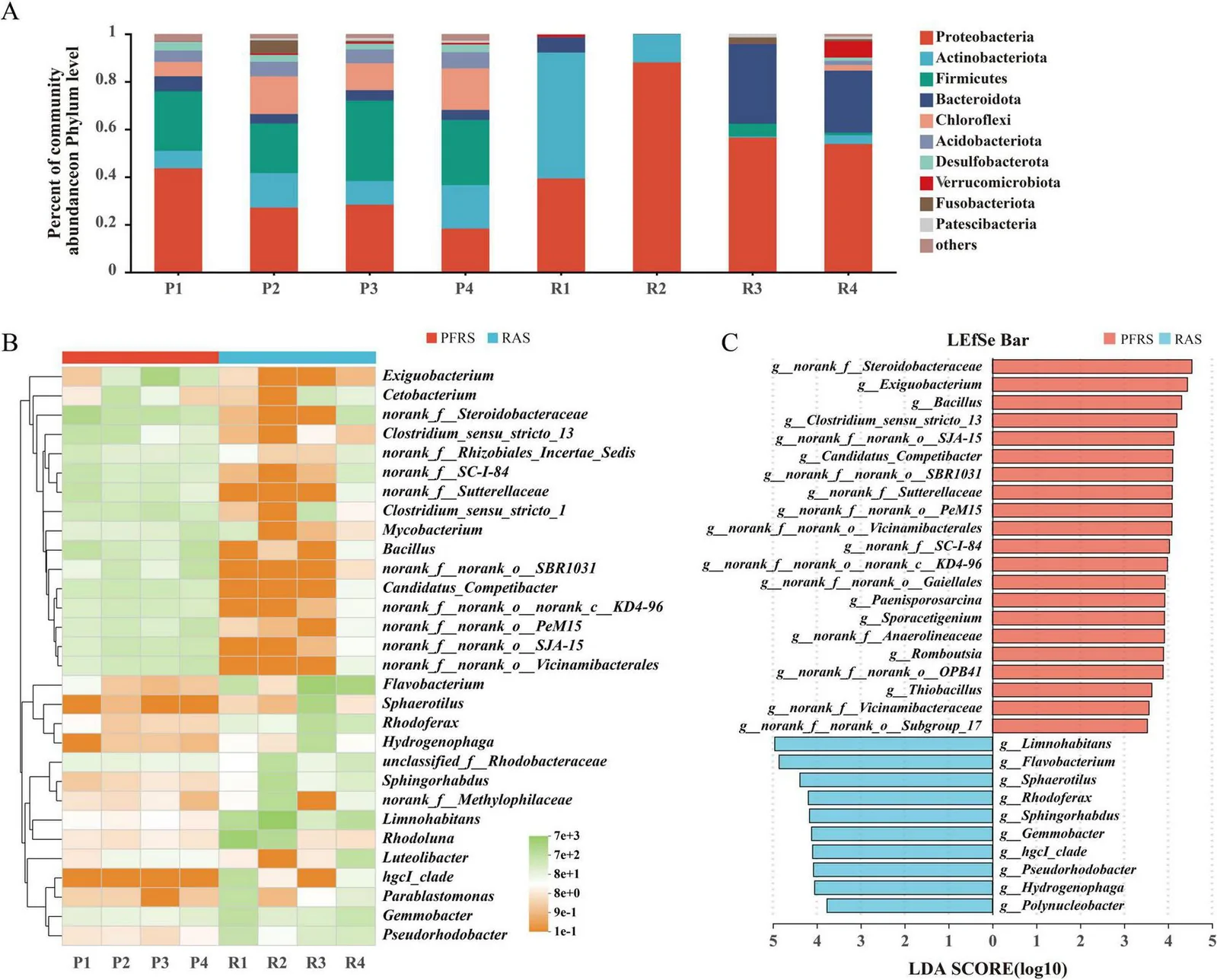
Bacterial community profiles and biomarkers between the PFRS and RAS groups. Phylum-level composition **(A)**, and genus-level abundance heatmap **(B)** are presented, LEfSe-identified differential taxa (LDA > 2) **(C)**.

As shown in Figure 4A, Proteobacteria, Actinobacteriota, and Firmicutes dominated across both systems but displayed significant differences in relative abundance at the phylum level. PFRS effluents were enriched in Firmicutes and Bacteroidota, whereas RAS effluents were characterized by higher proportions of Proteobacteria and Actinobacteriota. At the genus level, the abundance heatmap revealed clear system-dependent clustering of dominant bacterial taxa (Figure 4B). PFRS samples were mainly associated with higher abundances of *Exiguobacterium, Bacillus, Clostridium_sensu_stricto_13, Clostridium_sensu_stricto_1, Cetobacterium, Mycobacterium, Candidatus_Competibacter*, and several unclassified or norank lineages. In contrast, RAS samples were characterized by the enrichment of *Limnohabitans, Flavobacterium, Sphaerotilus, Rhodoferax, Hydrogenophaga, Sphingorhabdus, Rhodoluna, hgcI_clade, Gemmobacter*, and *Pseudorhodobacter*. This genus-level separation was consistent with the phylum-level differences shown in Figure 4A and indicated that cultivation mode reshaped bacterial communities from broad taxonomic composition to finer genus-level assemblages. The clustering pattern was also consistent with the LEfSe biomarkers in Figure 4C, supporting distinct microbial ecological signatures between PFRS and RAS effluents.

**FIGURE 5:**
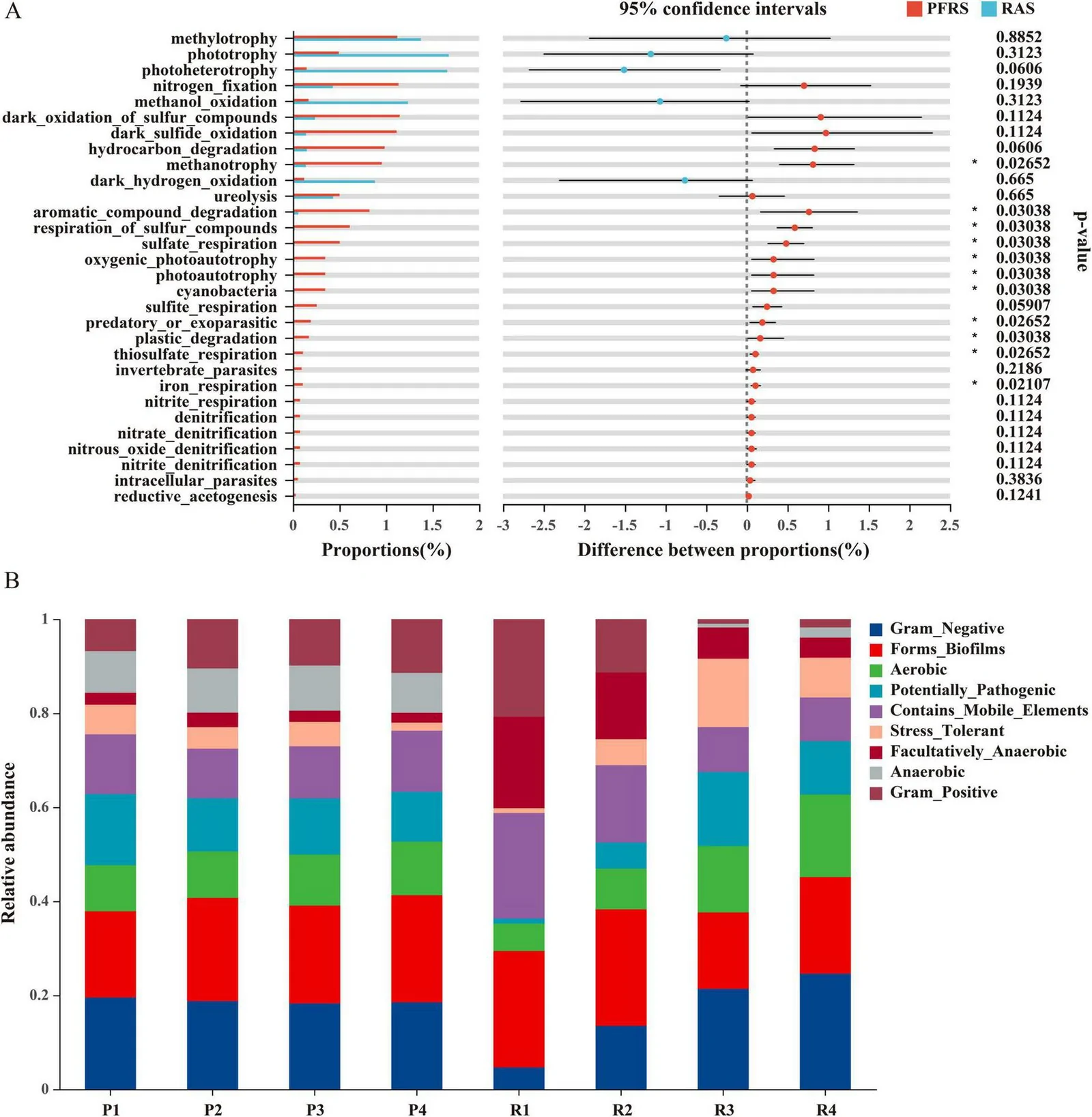
Functional **(A)** and phenotypic **(B)** predictions of bacterial communities in the PFRS and RAS groups. *Indicate statistically significant differences between the two groups (*p* < 0.05).

Community structural differentiation was mirrored by functional variation. Predicted functional pathways in RAS were enriched in nitrification, denitrification, and aromatic compound degradation, highlighting enhanced metabolic capacities associated with water purification and pollutant transformation (Figure 5A). BugBase phenotypic predictions indicated higher proportions of biofilm-forming and stress-tolerant phenotypes in RAS, reflecting adaptation to persistent selective pressures within closed-loop systems. Conversely, PFRS effluents showed enhanced fermentation-related pathways and higher proportions of predicted pathogenic phenotypes, consistent with adaptation to higher organic loading and relatively lower oxygen conditions (Figure 5B). The alignment between taxonomic shifts and functional predictions indicates coordinated structural and functional differentiation along environmental gradients.

### 3.3 RAS reduced conventional pollutants but did not reduce ARG and MGE burdens

**FIGURE 6:**
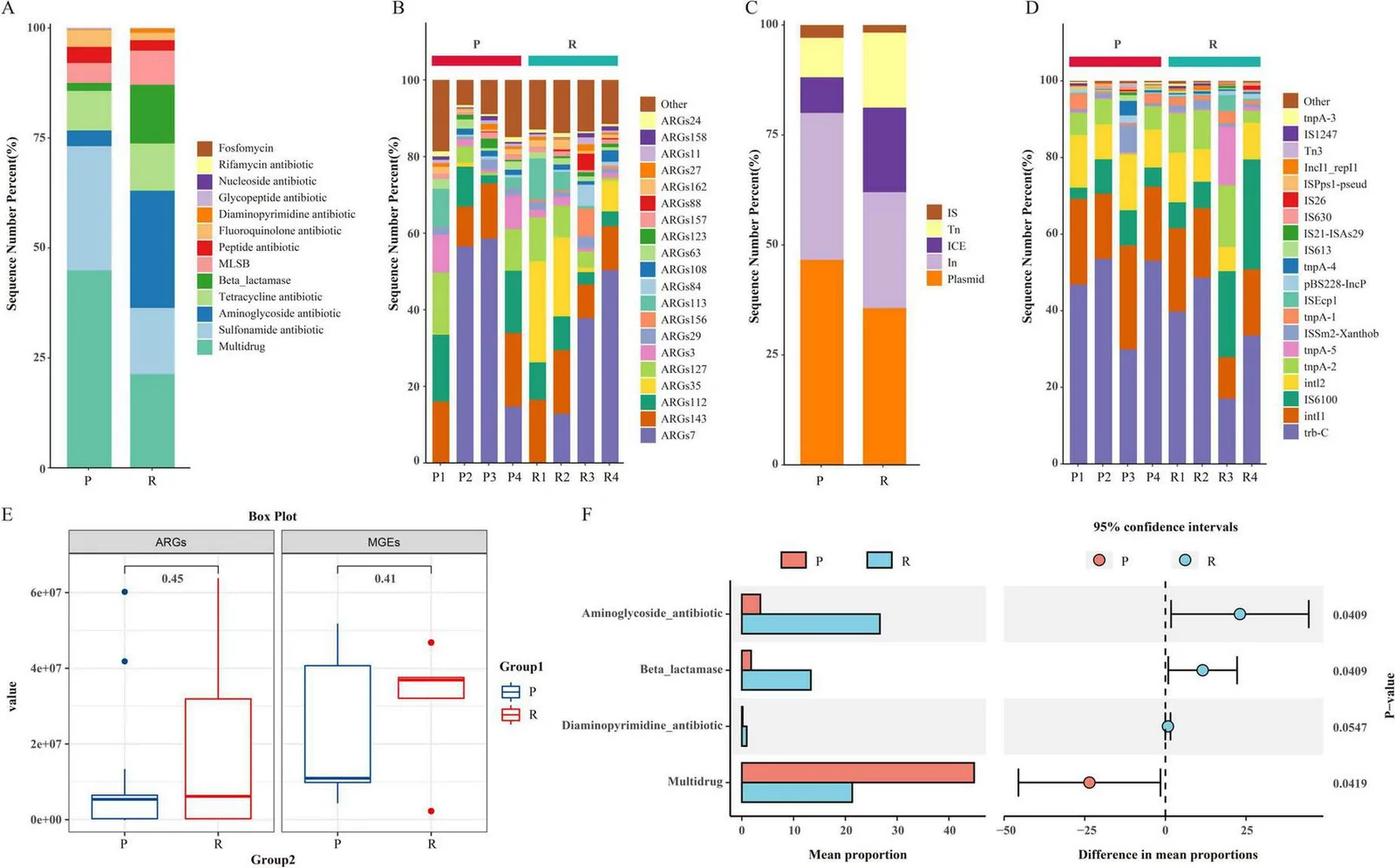
Profiles and comparative analysis of ARGs and MGEs between different aquaculture systems. Percentage distribution of ARGs sequences **(A)**; Relative abundance of ARGs **(B)**; Percentage distribution of MGEs sequences **(C)**; MGEs relative abundance **(D)**; ARGs-MGEs abundance variations **(E)**; Intergroup variation of microbial ARGs **(F)**.

At the ARG class level (Figure 6A and Supplementary Table 3), major resistance categories remained highly abundant in RAS effluents. At the ARG subtype level (Figure 6B and Supplementary Tables 3C,D), multiple resistance determinants exhibited distinct abundance patterns between cultivation modes. Several subtypes showed higher mean abundance in RAS, including blaSFO, tetA(P), dfrA1, dfrA17, tetJ, AAC(3)_Ib, CARB beta_lac, AAC(6)_Ib, lnuB, ErmF, tetM, tetB(P), lnuC, tetQ, EreB, and EreA, whereas other subtypes, including tetD, APH (4), vanA, QnrB4, AAC(3) _Via, and sul1, showed comparable or higher abundance in PFRS. Although several subtype-level comparisons showed large fold differences before correction, none remained significant after Benjamini–Hochberg adjustment (all BH q ≥ 0.670). Thus, subtype-level variation reflected differential distribution of individual resistance determinants rather than uniform enrichment across the entire ARG profile.

MGE profiles showed a similar pattern, with no evidence that the engineered purification process reduced genetic mobility-related markers (Figures 6C,D and Supplementary Tables 4A–C). Total MGE abundance was numerically higher in RAS than in PFRS (38.90 ± 23.18 × 10^6^ vs. 29.41 ± 13.02 × 10^6^), although the difference was not significant (*P* = 0.509). Several MGE subtypes, including IS6100, tnpA-2, tnpA-5, ISEcp1, IncN_rep, and IncI1_repI1, showed higher mean abundance in RAS, but none remained significant after FDR correction. Overall, RAS improved conventional effluent quality but did not reduce ARG or MGE burdens to the same extent.

Despite the substantial reduction of conventional pollutants in RAS effluents, ARG and MGE profiles were not concurrently reduced compared with PFRS (Figure 6E and Supplementary Tables 3A–C). Total ARG abundance was significantly higher in RAS than in PFRS, increasing from 35.39 ± 11.43 × 10^6^ to 63.99 ± 15.33 × 10^6^, corresponding to a 1.81-fold increase (*P* = 0.027). Aminoglycoside antibiotic resistance genes and β-lactamase genes showed the largest differences between systems, with 11.79-fold higher mean abundances in RAS than in PFRS (*P* = 0.029) (Figure 6F). However, these differences were not significant after Benjamini–Hochberg correction (BH *q* = 0.186). Diaminopyrimidine antibiotic resistance genes also showed higher abundance in RAS (10.36-fold), but the difference was not statistically significant (*P* = 0.086, BH *q* = 0.375). Other ARG classes showed variable distributions, with tetracycline, MLSB, peptide, rifamycin, and nucleoside resistance genes showing higher mean abundances in RAS, whereas multidrug, sulfonamide, fluoroquinolone, glycopeptide, and fosfomycin resistance genes were comparable or relatively higher in PFRS. This pattern indicates that improved removal of nitrogen, phosphorus, and organic matter was not accompanied by a proportional reduction in ARG burden.

### 3.4 Multilevel associations link environmental variation, microbial structure, and resistance-associated genetic elements

**FIGURE 7:**
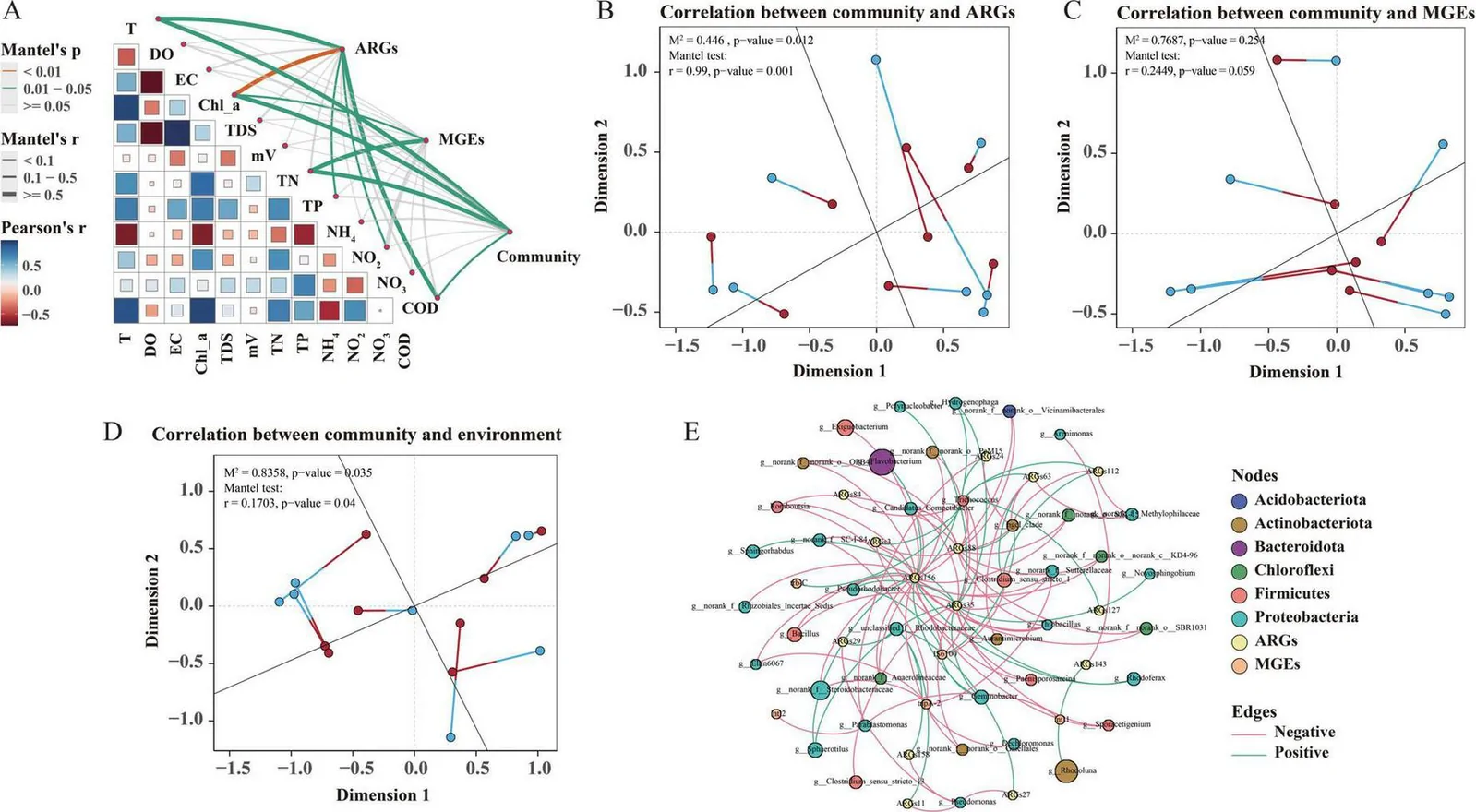
Linkages among microbial communities, environmental factors, ARGs, and MGEs. Mantel test showing correlations between microbial community composition, ARGs, MGEs, and environmental variables **(A)**; Procrustes analysis quantifying the structural concordance between microbial communities and environmental factors, ARGs, and MGEs, respectively **(B–D)**; Co-occurrence network analysis showing the interactions among bacterial taxa, ARGs, and MGEs **(E)**.

Mantel tests (Figure 7A) revealed that microbial community structure exhibited significant but selective correlations with environmental variables. Specifically, community structure was positively associated with TN, TP, COD, and Chl-a, whereas negative relationships were observed with DO, although not all correlations reached the same significance level. Overall, the results indicate that environmental gradients exert a measurable but not dominant influence on community assembly. ARGs showed a moderate but significant positive correlation with community structure (*P* = 0.001), suggesting a strong concordance between microbial composition and resistance gene profiles. In contrast, the relationship between community structure and MGEs was weak and marginally non-significant (*r* = 0.2449, *P* = 0.059), indicating that MGEs distribution is not tightly constrained by community composition. Meanwhile, MGEs were connected with ARGs in the co-occurrence network, indicating correlation-based associations among resistance-associated genetic elements rather than direct evidence of active gene transfer or dissemination.

Procrustes analysis further quantified these relationships. The community–environment fit was moderate (M^2^ = 0.8358, *P* = 0.035), indicating that environmental variables explained only part of the variation in microbial community structure. In contrast, the community–ARGs fit was substantially stronger (M^2^ = 0.446, *P* = 0.012), demonstrating a closer structural coupling between microbial communities and ARG profiles (Figure 7B). The community–MGEs fit was the weakest and statistically non-significant (M^2^ = 0.7687, *P* = 0.254) (Figure 7C), suggesting that MGEs are influenced by additional factors beyond community composition. These results support a hierarchical and partially decoupled relationship, in which environmental factors moderately shape microbial communities (Figure 7D), communities strongly correspond to ARGs structures, while MGE profiles showed weaker concordance with microbial community composition than ARG profiles, suggesting that their distribution may be influenced by factors not fully captured by community composition alone.

Co-occurrence network analysis revealed structured associations among microbial species, ARGs, and MGEs (Figure 7E and Supplementary Tables 5A–C). The constructed network consisted of 60 nodes and 96 edges, including 65 positive and 31 negative associations. Network density was 0.0542, with an average degree of 3.20. Louvain community detection identified 10 modules, with an overall modularity value (Q) of 0.4792 (Supplementary Table 5A). Network edges were defined based on significant Spearman correlations after Benjamini–Hochberg FDR correction (Supplementary Table 5B). Positive associations represented the predominant edge type, whereas negative associations were also observed among microbial species and resistance-associated genetic elements.

Centrality analysis identified highly connected nodes within the network (Supplementary Table 5C). Among ARG-associated nodes, tetB(P), blaSFO, and EreB exhibited high connectivity. TetB(P) showed the highest degree centrality among ARG nodes (degree = 26; degree centrality = 0.4407), followed by blaSFO (degree = 17; degree centrality = 0.2881) and EreB (degree = 12; degree centrality = 0.2034). Among MGE-associated nodes, IS6100 and tnpA-2 displayed relatively high connectivity. Several microbial species, including *g__Trichococcus, g__hgcI_clade*, and *g__Clostridium_sensu_stricto_1*, also exhibited high centrality within the network. Module assignment showed that ARGs, MGEs, and microbial species were distributed across multiple network modules. TetB(P), EreB, and associated microbial taxa were mainly assigned to one major module, whereas blaSFO, IS6100, and other resistance-associated elements were assigned to separate modules (Supplementary Table 5C). Overall, the co-occurrence network revealed a structured association pattern among microbial species, ARGs, and MGEs in aquaculture effluents. The identification of highly connected nodes and modular organization provided additional ecological context for the distribution patterns of resistance-associated genetic components.

## 4 Discussion

This study systematically compared water quality characteristics, microbial community structure and functional differentiation, as well as the distribution patterns of ARGs and MGEs in effluents from rainbow trout PFRS and RAS. The most theoretically significant and counterintuitive finding is that although RAS substantially reduced conventional pollution indicators, including TN, TP, COD, and Chl-a, it did not show a corresponding reduction in resistance-associated genetic indicators. Instead, total ARG abundance was significantly higher in RAS than in PFRS, while total MGE abundance showed no significant system-level difference. At the feature level, several ARG classes and subtypes maintained high absolute abundances or showed directional increases in RAS, although these responses were heterogeneous rather than uniformly confirmed across all individual features. This phenomenon challenges the conventional linear assumption that reduced pollutant loads necessarily correspond to lower resistance-associated genetic burden, suggesting that in engineered aquatic systems, traditional pollution control and genetic pollution control may operate under partially decoupled ecological processes. Integrating the multilevel association results identified in this study, we propose that ARG persistence in rainbow trout RAS reflects a multi-scale ecological process, in which environmental gradients are associated with microbial community restructuring, microbial community composition shows stronger correspondence with ARG profiles, and MGE profiles display weaker and more variable relationships with community composition rather than a clearly demonstrated system-wide enrichment.

### 4.1 RAS as an artificial microbial bioreactor: system-scale retention and ARG persistence

The engineering advantages of RAS, particularly in water conservation and nutrient removal, have been extensively documented (Fudge et al., 2023; Roy et al., 2025). Consistent with previous findings (Udayakumar et al., 2025), water quality measurements in this study confirmed significantly reduced concentrations of nitrogen, phosphorus, and organic matter in RAS effluents compared with PFRS. The multi-level ecological analyses showed that improvements in physicochemical water quality were not accompanied by a corresponding reduction in resistance-associated genetic indicators (Demeter et al., 2023). Specifically, total ARG abundance was higher in RAS, whereas total MGE abundance showed no significant system-level difference. This pattern suggests that ARG persistence in RAS is not fully explained by conventional pollutant loads, but is more closely associated with microbial community organization and resistance-related genetic profiles under recirculating conditions (Zheng et al., 2023; Huang et al., 2025).

From a systems perspective, the closed-loop configuration, low water exchange rate, and extended hydraulic retention time transform RAS from an open-flow aquatic environment into a continuously operating artificial microbial bioreactor (Ende et al., 2024). In PFRS, continuous inflow and discharge maintain an input, dilution, and output regime in which microbial populations and ARGs are largely regulated by hydrological flushing. In contrast, the recirculating operation of RAS prolongs microbial residence time and increases opportunities for microbial retention within water, biofilters, pipelines, suspended particles, and other system compartments. These conditions may favor the persistence of ARG-associated microbial assemblages and resistance-related genetic elements (Fang et al., 2024). Consequently, ARG persistence in RAS may shift from being mainly influenced by external hydrological inputs toward processes associated with internal retention, community stabilization, and selective ARG persistence (You et al., 2026), although this pattern appears heterogeneous across ARG classes and subtypes rather than uniform across the entire resistome.

Operational requirements for rainbow trout culture further reinforce this mechanism. Maintaining optimal growth conditions at 10 to 16 °C and high DO typically requires stable circulation with minimal hydraulic disturbance (Pepe-Victoriano et al., 2025), unintentionally reducing biomass export and promoting microbial stabilization within the system. Similar phenomena have been reported in engineered drinking-water distribution systems and advanced wastewater treatment facilities, where ARG persistence can occur despite substantial reductions in conventional pollutants, partly because of system enclosure, surface-associated habitats, and biofilm-mediated retention (Qu et al., 2025). These findings indicate that RAS effluents should not be interpreted solely as improved water quality outputs. Instead, RAS may function as a system-scale microbial bioreactor that reduces nutrient and organic pollutant loads while maintaining conditions favorable for ARG persistence over operational timescales (Bartelme et al., 2017), highlighting the necessity of integrating resistance-associated genetic indicators into conventional water-quality evaluation frameworks.

### 4.2 Community restructuring and niche selection under stable recirculating conditions

Conventional investigations of ARGs in aquaculture systems have primarily attributed resistance proliferation to direct selective pressures imposed by antibiotics or elevated nutrient loads (Wang et al., 2025). In contrast, the present study identified pronounced ARGs enrichment in RAS despite comparatively low pollutant concentrations, indicating that resistance persistence may be governed by alternative ecological mechanisms operating under recirculating conditions. Microbial community analyses showed lower alpha diversity in RAS than in PFRS, while PCoA and PERMANOVA demonstrated significant community differentiation between the two cultivation modes. Because PERMDISP was also significant, this separation should be interpreted as reflecting both compositional differences and altered within-system dispersion rather than a simple centroid shift alone. These results suggest that stable RAS operation reshaped effluent microbial assemblages into a more constrained and system-specific community structure.

Under the tightly regulated environmental conditions required for rainbow trout culture, characterized by stable temperature, DO, and water chemistry, RAS functions as a low-disturbance ecosystem (Pepe-Victoriano et al., 2025). In commercial RAS operation, biofilters are typically stabilized before fish culture begins, and the system is maintained under steady recirculating conditions during production. Therefore, the effluent communities observed in this study should be interpreted as microbial assemblages formed under stable recirculating operation, rather than as direct evidence of biofilter biofilm dynamics. Ecological theory predicts that reduced disturbance can suppress stochastic community replacement while promoting deterministic assembly processes, allowing taxa adapted to recurring system conditions to occupy relatively stable ecological niches (Shade et al., 2012; Bontemps et al., 2024) Within such stabilized environments, microbial communities may become increasingly shaped by internal system conditions rather than by short-term external inputs (Yang et al., 2026).

Once microbial taxa statistically associated with ARG profiles are retained within these recurring community structures, their associated resistance determinants may persist even without continuous external antibiotic input (Ben Maamar et al., 2020). Long-term persistence may therefore be supported by ecological inertia, microbial interaction networks, and repeated exposure to stable physicochemical conditions, rather than by direct antibiotic selection alone. This interpretation is consistent with the view that environmental AMR can be sustained by microbial community organization and ecological selection, not only by antibiotic residues or elevated nutrient loads (Larsson and Flach, 2022).

In contrast, PFRS experiences continuous hydrological disturbance and frequent microbial replacement, conditions that prevent long-term niche consolidation and promote dilution or physical removal of ARGs-associated taxa (Cheng et al., 2021). These contrasting dynamics suggest that the higher ARG burden observed in RAS may be associated with community restructuring and niche selection under stable recirculating operation, rather than with uniform enrichment driven solely by nutrient or antibiotic pressure (Zarean et al., 2026). Network analyses further support this interpretation by showing structured associations among microbial species, ARGs, and MGEs in aquaculture effluents, although these correlation-based associations cannot directly identify ARG hosts or demonstrate horizontal gene transfer. Consequently, conventional risk indicators, including antibiotic concentrations or instantaneous water-quality metrics, may underestimate resistance-associated genetic burden in engineered aquaculture systems.

### 4.3 Biofilm-associated microenvironments and possible co-selection in ARG persistence

Functional prediction analyses indicated an enhanced potential for biofilm formation in RAS compared with PFRS, suggesting that effluent microbial communities under recirculating conditions may contain a higher proportion of taxa with traits compatible with surface-associated growth. Biofilms are widely recognized as spatially structured microbial habitats in engineered aquatic systems, where cells are embedded in extracellular polymeric substances and exposed to microscale gradients in oxygen, nutrients, and chemical compounds (Stewart and Franklin, 2008; Flemming et al., 2016). In RAS, repeated water circulation, enclosed hydraulic conditions, and extensive wetted surfaces may increase opportunities for microbial retention and surface-associated persistence (Wang et al., 2026). These microenvironments can theoretically enhance cell-to-cell proximity and provide favorable conditions for genetic exchange processes such as conjugation, transposition, and integron-associated recombination (Aminov, 2011). Nevertheless, the present study analyzed outlet effluents rather than biofilter or pipeline biofilms directly. Therefore, the predicted biofilm-forming potential should be interpreted as an ecological trait signal associated with effluent communities, not as direct evidence that biofilm-mediated horizontal gene transfer occurred in the sampled systems. Similarly, although nitrifying and denitrifying microorganisms are central to RAS operation, the present data do not identify specific taxa as ARG hosts or exchange hubs. Thus, biofilm-associated microenvironments provide a plausible ecological background for ARG persistence in RAS, but they do not by themselves demonstrate an HGT-driven amplification mechanism.

Selection and co-selection may also contribute to ARG persistence, but the present evidence supports only an indirect interpretation. Sub-inhibitory antibiotic exposure can influence bacterial stress responses, mutation rates, and MGE-related activity (Revitt-Mills and Robinson, 2020), and bacterial diseases involving *Pseudomonas* spp., *Yersinia ruckeri*, and *Aeromonas hydrophila* have been reported in rainbow trout aquaculture systems (Nakatani et al., 2022). However, no prophylactic or therapeutic antibiotic use was recorded in the investigated farms during the documented pre-sampling period, and antibiotic residues, metals, disinfectants, and other potential co-selective agents were not directly measured. Therefore, historical or low-level selective pressure cannot be excluded, but it should not be treated as a confirmed driver of the observed ARG patterns. This interpretation is consistent with the MGE results: total ARG abundance was higher in RAS, whereas total MGE abundance showed no significant system-level enrichment, and individual MGE differences were not significant after FDR correction. Although integrons, transposons, and insertion sequences are important components of environmental resistance dissemination (Zhou et al., 2021), the co-occurrence network in this study revealed correlation-based associations among microbial species, ARGs, and MGEs rather than active gene capture, recombination, or diffusion processes. Accordingly, ARG persistence in RAS is better interpreted as being associated with system-scale retention, effluent microbial community organization, surface-associated ecological potential, and possible but unconfirmed selection or co-selection pressures, rather than with a directly demonstrated MGE-mediated or HGT-driven amplification pathway (Lin et al., 2021; Zhao et al., 2022; Liu et al., 2024).

### 4.4 Management implications, limitations, and future directions

**FIGURE 8:**
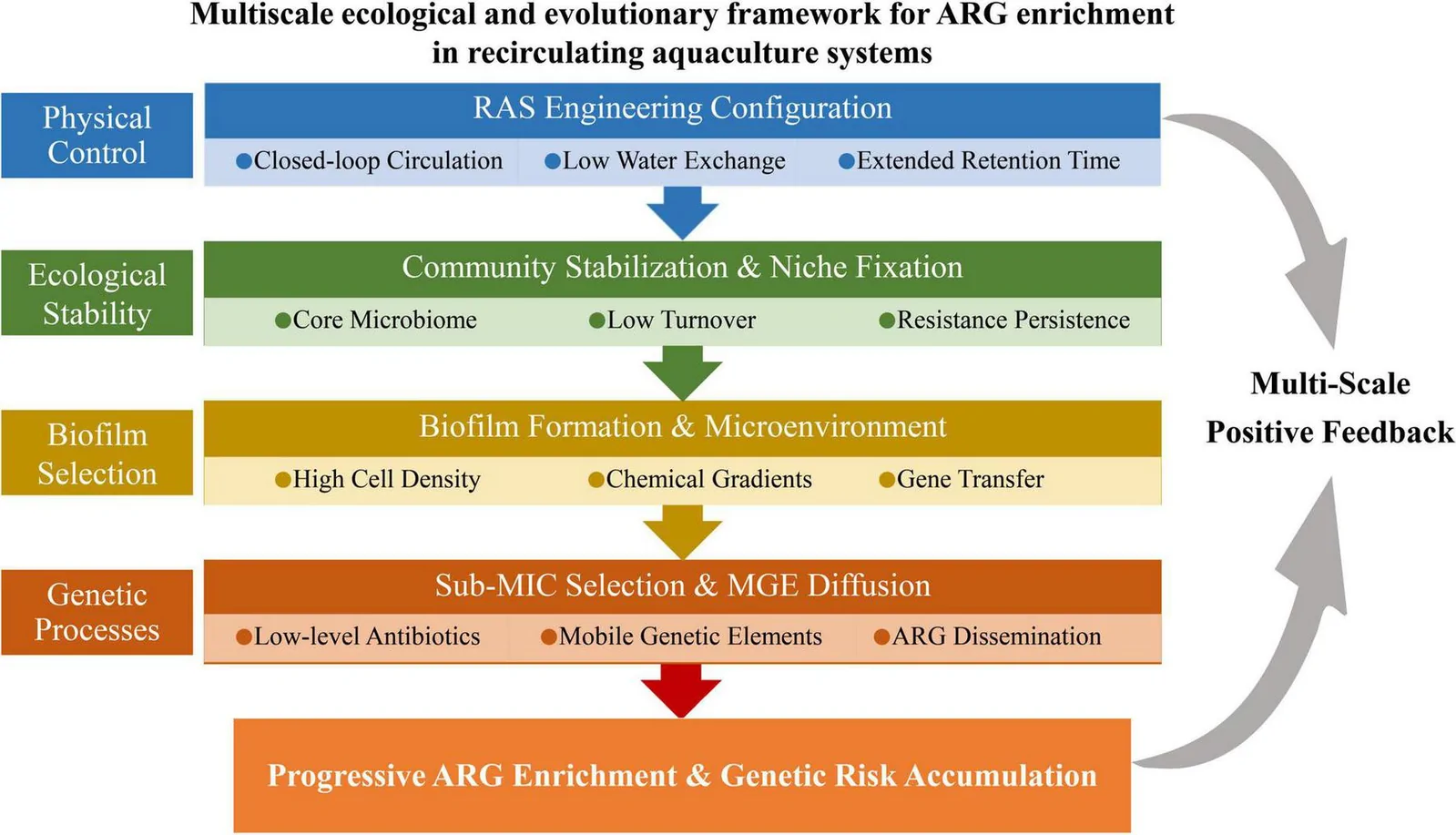
Conceptual framework illustrating potential ecological pathways associated with ARG persistence in RAS effluents.

Overall, the present findings suggest that ARG persistence in RAS should be understood within an integrated eco-engineering framework linking system design, microbial community organization, and resistance-associated genetic profiles (Figure 8). Unlike open-flow systems, RAS reduces nutrient and organic pollutant discharge through water reuse, solids removal, biological treatment, and operational stabilization. However, these same engineering features may also prolong the retention of microorganisms and genetic material, allowing resistance-associated profiles to persist under low-pollution conditions (Li et al., 2025). Therefore, the higher ARG burden observed in RAS does not necessarily indicate stronger external contamination, but rather suggests that conventional pollutant removal and resistance-associated genetic control may follow partially decoupled ecological processes. As aquaculture increasingly shifts toward high-density, low-exchange, land-based, and semi-closed production systems, management strategies should move beyond nutrient removal and operational efficiency alone (Baquero et al., 2011; Zhang et al., 2024).

From a broader eco-engineering perspective, these results highlight the need to expand conventional water-quality assessment toward a material–community–gene co-optimization framework. Traditional environmental engineering mainly regulates material fluxes, including nitrogen, phosphorus, organic matter, and energy, whereas ARGs represent biologically mediated genetic indicators that may persist through microbial community organization and gene-associated ecological processes even when pollutant concentrations decline (Zwanzig, 2020; Endale et al., 2023). Therefore, hydraulic retention time, water renewal frequency, solids removal, sludge handling, biofilter management, disinfection strategy, and feed inputs should be considered not only as water-quality control variables, but also as factors that may influence microbial and genetic retention. This broader view is consistent with the One Health perspective that aquaculture-associated antimicrobial resistance should be evaluated across environmental, animal, food, and management interfaces rather than through antibiotic use alone (Milijasevic et al., 2024). In this context, ARGs, MGEs, microbial community structure, and potential co-selective agents should be incorporated into routine RAS monitoring and predictive management, alongside TN, TP, COD, Chl-a, and DO.

These findings should be interpreted in light of several constraints inherent to field-scale investigations of commercial aquaculture systems. The number of eligible rainbow trout RAS farms was limited by the regional availability of large-scale recirculating facilities, although the present sampling design included all accessible systems that met the predefined criteria. Samples were collected at a single mid-cultivation stage, providing a field-scale snapshot of microbial community structure and resistance-associated genetic profiles under relatively stable production conditions. Therefore, longer-term temporal dynamics related to system start-up, seasonal fluctuation, stocking progression, or harvest-stage operation remain to be clarified. In addition, antibiotic residues, metals, disinfectants, microplastics, and other potential co-selective agents were not directly quantified in water, feed, sludge, biofilter compartments, sediments, or fish tissues. Thus, antibiotic-related selection and co-selection cannot be confirmed as direct drivers, although they remain relevant factors for future investigation. Similarly, the combined use of 16S rRNA gene sequencing, GeoChip profiling, and correlation-based network analysis provided community- and gene-level association patterns, but could not directly resolve ARG host identity, plasmid-mediated mobility, active horizontal gene transfer, or transcriptional activity.

Despite these constraints, the present study provides field-based evidence that improved conventional water quality in RAS does not necessarily correspond to reduced ARG burden in effluents. This finding has important implications for sustainable aquaculture management and One Health-oriented AMR surveillance, because aquaculture effluents represent an environmental interface between intensive production systems and surrounding aquatic ecosystems. Future studies integrating longitudinal sampling, chemical residue analysis, fish-tissue assessment, downstream monitoring, and genome-resolved approaches will help clarify exposure pathways, host–gene linkages, and mobility mechanisms, thereby strengthening public-health-oriented risk assessment and management of RAS effluents. Future studies integrating metagenomic, genome-resolved, and long-read sequencing approaches, together with methods capable of linking ARGs to microbial hosts and MGEs, would help clarify host–ARG–MGE relationships and mobility potential (Tian et al., 2024). Overall, sustainable RAS design should aim not only to reduce pollutant discharge, but also to balance water-quality control, functional microbial stability, and mitigation of ARG-associated ecological risk.

## 5 Conclusion

This study demonstrated that RAS substantially improved conventional effluent quality in commercial rainbow trout aquaculture, as indicated by lower TN, TP, COD, and Chl-a concentrations compared with PFRS. However, this improvement did not correspond to a proportional reduction in resistance-associated genetic indicators. Total ARG abundance was significantly higher in RAS effluents, whereas total MGE abundance showed no significant system-level difference, revealing a decoupling between conventional pollutant removal and ARG burden under recirculating conditions. Microbial community analyses further showed that RAS effluents harbored less diverse and compositionally distinct bacterial assemblages, suggesting that recirculating operation reshaped effluent microbial communities under low-pollution conditions. ARG profiles were more closely associated with microbial community structure than MGE profiles, and co-occurrence network analysis identified structured associations among microbial species, ARGs, and MGEs. These findings suggest that ARG persistence in RAS may be linked to microbial community restructuring, system-scale retention, and resistance-associated ecological organization, rather than being fully explained by conventional water-quality indicators alone. The results should be interpreted within the scope of field-scale effluent monitoring, as this study did not directly resolve ARG host identity, active horizontal gene transfer, food-chain exposure, consumer risk, or downstream public-health impact. Nevertheless, the detection of elevated ARG burden in low-pollution RAS effluents has important implications for sustainable aquaculture management and One Health-oriented AMR surveillance. Future studies integrating longitudinal sampling, chemical residue analysis, fish-tissue assessment, downstream monitoring, and genome-resolved approaches are needed to clarify host–ARG–MGE linkages and broader exposure pathways. Overall, sustainable RAS management should extend beyond conventional water-quality assessment by incorporating ARGs, MGEs, microbial community structure, and potential co-selective agents into routine monitoring and risk evaluation frameworks.

## Data Availability

The original contributions presented in the study are publicly available. These data can be found in the NCBI Sequence Read Archive (SRA) under BioProject accession number PRJNA1496462.
