# Supplementary material for: Unexpected enrichment of antibiotic resistance genes in recirculating aquaculture effluents of rainbow trout (Oncorhynchus mykiss): microbial community assembly and mobile genetic elements as key drivers
Source: Front Microbiol. 2026 Aug 12;17:1913067. doi: 10.3389/fmicb.2026.1913067 (PMC13506868; doi:10.3389/fmicb.2026.1913067)
Supplement: Supplementary file 1 [file Data_Sheet_1.pdf]

Table S1 Integrated farm-level feed, antibiotic-use, disease-management, and operational records

Farm_ID	System	Region / water / sampling	Fish and stocking	Feed composition and feeding	Feed additives and feed-derived tests	Antibiotic and disease-management records	Disinfectants, residues, and co-selective agents	Operational parameters	RAS/PFRS infrastructure	Sampling design and evidence
PFRS-1	PFRS	Bai Shan; spring water; May-25 11:00	Fish weight: 495.21 ± 25.75 g; stocking density: 12.77 ± 0.75 kg/m3	Hanye; Commercial extruded feed; pellet 5 mm; crude protein 40%; lipid 12%; moisture 10%; ash 14%; fiber 5%; feeding rate 1.08±0.75% BW/day; FCR/recorded feed index: 83	Feed changed within 30 d: No; medicated feed: No; probiotic additives: No; antibiotic growth promoter: No; immune/bioactive additives: No; other additives: No; feed microbiome/residue/metal/biocide tests: Not measured	90-d record lookback; routine prophylactic antibiotic use: No; therapeutic antibiotic use: No; antibiotic name/dose/route/last use: Not applicable; withdrawal period: Not applicable; major disease outbreak: No; emergency treatment: No	Disinfectant/biocide use recorded in disease-management table: No; metal/mineral additive: No; antibiotic residue measured: No; residue sampling: Not measured; reason: commercial farms did not permit additional residue sampling during this field investigation	Temperature: 13.27 ± 0.35 C; DO: 7.84 ± 0.12 mg/L; pH: 7.63 ± 0.12; flow: 90 m3/h; water exchange: 7200%/day; hydraulic retention time: 0.33 h	Biofilter: No; media: No; biofilm/backwash: No; disinfection strategy: No; solids removal: No	No major disease outbreak: Yes; no emergency treatment: Yes; routine management consistent: Yes; composite sampling used: Yes; farm as biological replicate: Yes; evidence: production log / manager interview
PFRS-2	PFRS	Bai Shan; spring water; May-25 10:00	Fish weight: 494.88 ± 23.75 g; stocking density: 12.33 ± 0.76 kg/m3	Hanye; Commercial extruded feed; pellet 5 mm; crude protein 40%; lipid 12%; moisture 10%; ash 14%; fiber 5%; feeding rate 1.08±0.03% BW/day; FCR/recorded feed index: 81	Feed changed within 30 d: No; medicated feed: No; probiotic additives: No; antibiotic growth promoter: No; immune/bioactive additives: No; other additives: No; feed microbiome/residue/metal/biocide tests: Not measured	90-d record lookback; routine prophylactic antibiotic use: No; therapeutic antibiotic use: No; antibiotic name/dose/route/last use: Not applicable; withdrawal period: Not applicable; major disease outbreak: No; emergency treatment: No	Disinfectant/biocide use recorded in disease-management table: No; metal/mineral additive: No; antibiotic residue measured: No; residue sampling: Not measured; reason: commercial farms did not permit additional residue sampling during this field investigation	Temperature: 13.27 ± 0.42 C; DO: 8.17 ± 0.31 mg/L; pH: 7.67 ± 0.06; flow: 90 m3/h; water exchange: 7200%/day; hydraulic retention time: 0.33 h	Biofilter: No; media: No; biofilm/backwash: No; disinfection strategy: No; solids removal: No	No major disease outbreak: Yes; no emergency treatment: Yes; routine management consistent: Yes; composite sampling used: Yes; farm as biological replicate: Yes; evidence: production

										log / manager interview
PFRS-3	PFRS	Dandong; spring water; May-25 9:00	Fish weight: 496.42 ± 23.64 g; stocking density: 13.33 ± 0.58 kg/m3	Hanye; Commercial extruded feed; pellet 5 mm; crude protein 40%; lipid 12%; moisture 10%; ash 14%; fiber 5%; feeding rate 1.07±0.06% BW/day; FCR/recorded feed index: 83	Feed changed within 30 d: No; medicated feed: No; probiotic additives: No; antibiotic growth promoter: No; immune/bioactive additives: No; other additives: No; feed microbiome/residue/metal/biocide tests: Not measured	90-d record lookback; routine prophylactic antibiotic use: No; therapeutic antibiotic use: No; antibiotic name/dose/route/last use: Not applicable; withdrawal period: Not applicable; major disease outbreak: No; emergency treatment: No	Disinfectant/biocide use recorded in disease-management table: No; metal/mineral additive: No; antibiotic residue measured: No; residue sampling: Not measured; reason: commercial farms did not permit additional residue sampling during this field investigation	Temperature: 13.93 ± 0.40 C; DO: 8.47 ± 0.21 mg/L; pH: 7.57 ± 0.06; flow: 30 m3/h; water exchange: 7200%/day; hydraulic retention time: 0.33 h	Biofilter: No; media: No; biofilm/backwash: No; disinfection strategy: No; solids removal: No	No major disease outbreak: Yes; no emergency treatment: Yes; routine management consistent: Yes; composite sampling used: Yes; farm as biological replicate: Yes; evidence: production log / manager interview
PFRS-4	PFRS	Dandong; spring water; May-25 10:00	Fish weight: 497.04 ± 24.81 g; stocking density: 11.83 ± 1.04 kg/m3	Hanye; Commercial extruded feed; pellet 5 mm; crude protein 40%; lipid 12%; moisture 10%; ash 14%; fiber 5%; feeding rate 0.97±0.06% BW/day; FCR/recorded feed index: 82	Feed changed within 30 d: No; medicated feed: No; probiotic additives: No; antibiotic growth promoter: No; immune/bioactive additives: No; other additives: No; feed microbiome/residue/metal/biocide tests: Not measured	90-d record lookback; routine prophylactic antibiotic use: No; therapeutic antibiotic use: No; antibiotic name/dose/route/last use: Not applicable; withdrawal period: Not applicable; major disease outbreak: No; emergency treatment: No	Disinfectant/biocide use recorded in disease-management table: No; metal/mineral additive: No; antibiotic residue measured: No; residue sampling: Not measured; reason: commercial farms did not permit additional residue sampling during this field investigation	Temperature: 13.10 ± 0.40 C; DO: 8.11 ± 0.09 mg/L; pH: 7.67 ± 0.12; flow: 30 m3/h; water exchange: 7200%/day; hydraulic retention time: 0.33 h	Biofilter: No; media: No; biofilm/backwash: No; disinfection strategy: No; solids removal: No	No major disease outbreak: Yes; no emergency treatment: Yes; routine management consistent: Yes; composite sampling used: Yes; farm as biological replicate: Yes; evidence: production log / manager interview
RAS-1	RAS	Dalian; groundwate	Fish weight: 512.92 ±	Hanye; Commercial extruded feed;	Feed changed within 30 d: No; medicated feed: No; probiotic additives: No;	90-d record lookback; routine prophylactic	Disinfectant/biocide use recorded in disease-	Temperature: 14.30 ± 0.20 C; DO: 7.71 ±	Biofilter: Moving Bed Biofilm Reactor; media:	No major disease outbreak:

		r; Mar-25 10:00	26.86 g; stocking density: 25.17 ± 1.26 kg/m3	pellet 5 mm; crude protein 40%; lipid 12%; moisture 10%; ash 14%; fiber 5%; feeding rate 1.33±0.06% BW/day; FCR/recorded feed index: 91	antibiotic growth promoter: No; immune/bioactive additives: No; other additives: No; feed microbiome/residue/metal/biocide tests: Not measured	antibiotic use: No; therapeutic antibiotic use: No; antibiotic name/dose/route/last use: Not applicable; withdrawal period: Not applicable; major disease outbreak: No; emergency treatment: No	management table: No; metal/mineral additive: No; antibiotic residue measured: No; residue sampling: Not permitted for additional residue sampling; reason: commercial farms did not permit additional residue sampling during this field investigation	0.31 mg/L; pH: 7.47 ± 0.06; flow: 200 m3/h; water exchange: 9600%/day; hydraulic retention time: 0.25	high-density polyethylene; biofilm/backwash: No backwash required; disinfection strategy: ultraviolet and ozone; solids removal: Drum Microfilter and Foam Fractionator	Yes; no emergency treatment: Yes; routine management consistent: Yes; composite sampling used: Yes; farm as biological replicate: Yes; evidence: production log / manager interview
RAS-2	RAS	Dalian; groundwater; Mar-25 12:00	Fish weight: 516.04 ± 22.11 g; stocking density: 25.67 ± 1.53 kg/m3	Hanye; Commercial extruded feed; pellet 5 mm; crude protein 40%; lipid 12%; moisture 10%; ash 14%; fiber 5%; feeding rate 1.32±0.08% BW/day; FCR/recorded feed index: 90	Feed changed within 30 d: No; medicated feed: No; probiotic additives: No; antibiotic growth promoter: No; immune/bioactive additives: No; other additives: No; feed microbiome/residue/metal/biocide tests: Not measured	90-d record lookback; routine prophylactic antibiotic use: No; therapeutic antibiotic use: No; antibiotic name/dose/route/last use: Not applicable; withdrawal period: Not applicable; major disease outbreak: No; emergency treatment: No	Disinfectant/biocide use recorded in disease-management table: No; metal/mineral additive: No; antibiotic residue measured: No; residue sampling: Not permitted for additional residue sampling; reason: commercial farms did not permit additional residue sampling during this field investigation	Temperature: 12.87 ± 0.35 C; DO: 8.67 ± 0.44 mg/L; pH: 7.57 ± 0.12; flow: 200 m3/h; water exchange: 9600%/day; hydraulic retention time: 0.25 h	Biofilter: Moving Bed Biofilm Reactor; media: high-density polyethylene; biofilm/backwash: No backwash required; disinfection strategy: ultraviolet and ozone; solids removal: Drum Microfilter and Foam Fractionator	No major disease outbreak: Yes; no emergency treatment: Yes; routine management consistent: Yes; composite sampling used: Yes; farm as biological replicate: Yes; evidence: production log / manager interview
RAS-3	RAS	Weihai; groundwater; Mar-25 10:00	Fish weight: 508.21 ± 26.60 g; stocking density: 26.67 ± 1.53 kg/m3	Hanye; Commercial extruded feed; pellet 5 mm; crude protein 40%; lipid 12%; moisture 10%; ash 14%; fiber 5%;	Feed changed within 30 d: No; medicated feed: No; probiotic additives: No; antibiotic growth promoter: No; immune/bioactive additives: No; other additives: No; feed microbiome/residue/metal/biocide tests: Not measured	90-d record lookback; routine prophylactic antibiotic use: No; therapeutic antibiotic use: No; antibiotic name/dose/route/last use: Not	Disinfectant/biocide use recorded in disease-management table: No; metal/mineral additive: No; antibiotic residue measured: No;	Temperature: 12.93 ± 0.15 C; DO: 9.63 ± 0.91 mg/L; pH: 7.53 ± 0.06; flow: 400 m3/h; water exchange: 9600%/day;	Biofilter: Moving Bed Biofilm Reactor; media: high-density polyethylene; biofilm/backwash: No backwash required; disinfection	No major disease outbreak: Yes; no emergency treatment: Yes; routine managemen

				feeding rate 1.33±0.06% BW/day; FCR/recorded feed index: 90		applicable; withdrawal period: Not applicable; major disease outbreak: No; emergency treatment: No	residue sampling: Not permitted for additional residue sampling; reason: commercial farms did not permit additional residue sampling during this field investigation	hydraulic retention time: 0.25 h	strategy: ultraviolet and ozone; solids removal: Drum Microfilter and Foam Fractionator	t consistent: Yes; composite sampling used: Yes; farm as biological replicate: Yes; evidence: production log / manager interview
RAS-4	RAS	Weihai; groundwater; Mar-25 11:00	Fish weight: 511.75 ± 19.47 g; stocking density: 25.83 ± 1.61 kg/m3	Hanye; Commercial extruded feed; pellet 5 mm; crude protein 40%; lipid 12%; moisture 10%; ash 14%; fiber 5%; feeding rate 1.28±0.08% BW/day; FCR/recorded feed index: 90	Feed changed within 30 d: No; medicated feed: No; probiotic additives: No; antibiotic growth promoter: No; immune/bioactive additives: No; other additives: No; feed microbiome/residue/metal/biocide tests: Not measured	90-d record lookback; routine prophylactic antibiotic use: No; therapeutic antibiotic use: No; antibiotic name/dose/route/last use: Not applicable; withdrawal period: Not applicable; major disease outbreak: No; emergency treatment: No	Disinfectant/biocide use recorded in disease-management table: No; metal/mineral additive: No; antibiotic residue measured: No; residue sampling: Not permitted for additional residue sampling; reason: commercial farms did not permit additional residue sampling during this field investigation	Temperature: 12.63 ± 0.15 C; DO: 9.67 ± 0.67 mg/L; pH: 7.57 ± 0.06; flow: 400 m3/h; water exchange: 9600%/day; hydraulic retention time: 0.25 h	Biofilter: Moving Bed Biofilm Reactor; media: high-density polyethylene; biofilm/backwash: No backwash required; disinfection strategy: ultraviolet and ozone; solids removal: Drum Microfilter and Foam Fractionator	No major disease outbreak: Yes; no emergency treatment: Yes; routine management consistent: Yes; composite sampling used: Yes; farm as biological replicate: Yes; evidence: production log / manager interview

Table S2A. 16S sequencing quality-control summary for each sample

SampleInfo	System	Seq_num	Base_num	Mean_length	Min_length	Max_length
R1	RAS	57256	23781522	415.35423	297	431
R2	RAS	43671	18375375	420.76836	308	447
R3	RAS	56411	23886762	423.44156	210	519
R4	RAS	50795	21051078	414.43209	281	478
P1	PFRS	49429	20779538	420.39163	301	447
P2	PFRS	50785	21168272	416.82135	246	523
P3	PFRS	51476	21596822	419.55129	246	478
P4	PFRS	45333	18832641	415.42896	291	458

Table S2B. Alpha-diversity comparisons between RAS and PFRS effluent communities

Metric	RAS mean ± SD	PFRS mean ± SD	RAS/PFRS fold	P value	Hedges' g	95% CI of fold	Direction
Sobs	203.75 ± 166.97	561.75 ± 32.79	0.36	0.021	-2.59	0.18-0.66	PFRS > RAS
Chao1	239.85 ± 181.60	590.05 ± 24.82	0.41	0.030	-2.35	0.22-0.71	PFRS > RAS
Shannon	3.10 ± 1.06	5.02 ± 0.31	0.62	0.031	-2.14	0.46-0.81	PFRS > RAS
Simpson	0.124 ± 0.081	0.020 ± 0.015	5.04	0.089	1.48	2.08-12.30	RAS > PFRS
Coverage	0.997 ± 0.002	0.995 ± 0.001	1.00	0.218	0.89	1.00-1.00	RAS > PFRS
Pielou's evenness	0.595 ± 0.118	0.793 ± 0.047	0.75	0.037	-1.92	0.63-0.89	PFRS > RAS

Table S2C. PERMANOVA and PERMDISP

Dataset	Transformation	Distance	PERMANOVA_pseudo_F	PERMANOVA_R2	PERMANOVA_P_value	PERMANOVA_permutations	PERMDISP_F	PERMDISP_P_value	PERMDISP_permutations	Mean_distance_to_centroid_RAS	Mean_distance_to_centroid_PFRS
16S OTU community	relative abundance	Bray-Curtis	4.287139405480456	0.41674747823447306	0.028	999	66.2770295068841 6	0.028	999	0.5066147758502836	0.2920797684648339 7

Table S2C. Top 30 bacterial taxa by total abundance

phylum	class	order	family	genus	Total	Prevalence	Percent
p__Proteobacteria	c__Gammaproteobacteria	o__Burkholderiales	f__Comamonadaceae	g__Limnohabitans	6433	0.75	0.066243101
p__Actinobacteriota	c__Actinobacteria	o__Micrococcales	f__Microbacteriaceae	g__Rhodoluna	5858	1.0	0.060322102
p__Proteobacteria	c__Gammaproteobacteria	o__Steroidobacterales	f__Steroidobacteraceae	g__norank_f__Steroidobacteraceae	3662	0.625	0.037709037
p__Firmicutes	c__Bacilli	o__Exiguobacterales	f__Exiguobacteraceae	g__Exiguobacterium	2551	0.625	0.026268638
p__Proteobacteria	c__Gammaproteobacteria	o__Burkholderiales	f__Comamonadaceae	g__Sphaerotilus	2322	0.625	0.023910536
p__Proteobacteria	c__Alphaproteobacteria	o__Rhodobacterales	f__Rhodobacteraceae	g__Gemmobacter	2003	1.0	0.020625669
p__Bacteroidota	c__Bacteroidia	o__Flavobacteriales	f__Flavobacteriaceae	g__Flavobacterium	2002	0.5	0.020615372

p__Bacteroidota	c__Bacteroidia	o__Flavobacteriales	f__Flavobacteriaceae	g__Flavobacterium	1839	0.375	0.018936898
p__Bacteroidota	c__Bacteroidia	o__Flavobacteriales	f__Flavobacteriaceae	g__Flavobacterium	1668	0.75	0.017176044
p__Proteobacteria	c__Alphaproteobacteria	o__Sphingomonadales	f__Sphingomonadaceae	g__Sphingorhabdus	1618	1.0	0.016661175
p__Firmicutes	c__Bacilli	o__Bacillales	f__Bacillaceae	g__Bacillus	1546	0.75	0.015919763
p__Proteobacteria	c__Gammaproteobacteria	o__Burkholderiales	f__Comamonadaceae	g__Limnohabitans	1439	0.5	0.014817942
p__Firmicutes	c__Clostridia	o__Clostridiales	f__Clostridiaceae	g__Clostridium_sensu_stricto_13	1411	0.875	0.014529615
p__Proteobacteria	c__Gammaproteobacteria	o__Burkholderiales	f__Methylophilaceae	g__norank_f__Methylophilaceae	1255	0.375	0.012923223
p__Proteobacteria	c__Gammaproteobacteria	o__Burkholderiales	f__Comamonadaceae	g__Hydrogenophaga	1149	0.875	0.011831699
p__Proteobacteria	c__Gammaproteobacteria	o__Burkholderiales	f__Comamonadaceae	g__Rhodoferax	1082	0.875	0.011141774
p__Actinobacteriota	c__Actinobacteria	o__Frankiales	f__Sporichthyaceae	g__hgcI_clade	1046	0.375	0.010771068
p__Proteobacteria	c__Gammaproteobacteria	o__Competibacterales	f__Competibacteraceae	g__Candidatus_Competibacter	962	0.625	0.009906088
p__Proteobacteria	c__Alphaproteobacteria	o__Sphingomonadales	f__Sphingomonadaceae	g__Parablastomonas	960	0.875	0.009885493
p__Proteobacteria	c__Alphaproteobacteria	o__Rhizobiales	f__Rhizobiales_Incertae_Sedis	g__norank_f__Rhizobiales_Incertae_Sedis	944	1.0	0.009720735
p__Proteobacteria	c__Alphaproteobacteria	o__Rhodobacterales	f__Rhodobacteraceae	g__unclassified_f__Rhodobacteraceae	916	0.875	0.009432408
p__Proteobacteria	c__Gammaproteobacteria	o__Burkholderiales	f__SC-I-84	g__norank_f__SC-I-84	891	0.875	0.009174973
p__Actinobacteriota	c__Actinobacteria	o__Micrococcales	f__Microbacteriaceae	g__Aurantimicrobium	867	0.875	0.008927836
p__Proteobacteria	c__Alphaproteobacteria	o__Rhodobacterales	f__Rhodobacteraceae	g__Pseudorhodobacter	853	1.0	0.008783672
p__Firmicutes	c__Bacilli	o__Bacillales	f__Planococcaceae	g__Paenisporosarcina	831	0.625	0.00855713
p__Firmicutes	c__Clostridia	o__Peptostreptococcales-Tissierellales	f__Peptostreptococcaceae	g__Sporacetigenium	776	0.5	0.007990774
p__Verrucomicrobiota	c__Verrucomicrobiae	o__Verrucomicrobiales	f__Rubritaleaceae	g__Luteolibacter	770	0.5	0.007928989
p__Proteobacteria	c__Gammaproteobacteria	o__Burkholderiales	f__Comamonadaceae	g__Limnohabitans	733	0.5	0.007547986
p__Firmicutes	c__Clostridia	o__Peptostreptococcales-Tissierellales	f__Peptostreptococcaceae	g__Romboutsia	723	0.75	0.007445012
p__Firmicutes	c__Clostridia	o__Clostridiales	f__Clostridiaceae	g__Clostridium_sensu_stricto_1	690	0.625	0.007105198

Table S3A. Total ARG abundance comparison between RAS and PFRS

Metric	RAS mean ± SD	PFRS mean ± SD	RAS/PFRS fold	P value
ARG total abundance	$63.99 \times 10^6 \pm 15.33 \times 10^6$	$35.39 \times 10^6 \pm 11.43 \times 10^6$	1.81	0.027

Table S3B. ARG class-level abundance comparisons between RAS and PFRS

Feature	RAS mean ± SD	PFRS mean ± SD	Fold	P	BH q	Direction
Aminoglycoside antibiotic	$15.95 \times 10^6 \pm 7.52 \times 10^6$	$1.35 \times 10^6 \pm 1.18 \times 10^6$	11.79	0.029	0.186	RAS > PFRS
Beta_lactamase	$7.98 \times 10^6 \pm 3.76 \times 10^6$	676,849 ± 589,200	11.79	0.029	0.186	RAS > PFRS
Diaminopyrimidine antibiotic	646,758 ± 466,080	62,418 ± 46,781	10.36	0.086	0.375	RAS > PFRS
Fluoroquinolone antibiotic	1.09×10^6 ± 502,627	1.38×10^6 ± 872,987	0.79	0.590	0.698	PFRS > RAS
MLSB	$5.71 \times 10^6 \pm 8.26 \times 10^6$	1.62×10^6 ± 579,736	3.54	0.395	0.698	RAS > PFRS
Glycopeptide antibiotic	61,782 ± 39,571	98,760 ± 75,897	0.63	0.431	0.698	PFRS > RAS
Multidrug	$13.38 \times 10^6 \pm 2.45 \times 10^6$	$15.05 \times 10^6 \pm 4.71 \times 10^6$	0.89	0.560	0.698	PFRS > RAS
Tetracycline antibiotic	$7.69 \times 10^6 \pm 7.44 \times 10^6$	$3.31 \times 10^6 \pm 2.42 \times 10^6$	2.33	0.331	0.698	RAS > PFRS
Peptide antibiotic	1.54×10^6 ± 763,791	1.34×10^6 ± 796,868	1.15	0.732	0.793	RAS > PFRS
Sulfonamide antibiotic	$9.87 \times 10^6 \pm 4.16 \times 10^6$	$10.46 \times 10^6 \pm 6.22 \times 10^6$	0.94	0.880	0.880	PFRS > RAS
Rifamycin antibiotic	24,243 ± 33,770	5,009 ± 5,809	4.84	0.339	0.698	RAS > PFRS
Fosfomycin	5,197 ± 3,795	26,200 ± 20,519	0.20	0.132	0.428	PFRS > RAS
Nucleoside antibiotic	35,214 ± 45,819	17,230 ± 20,438	2.04	0.512	0.698	RAS > PFRS

Table S3C. ARG subtype abundance comparison between RAS and PFRS

Gene	P1	P2	P3	P4	R1	R2	R3	R4	Code
APH(3)	10,175	0	0	0	0	0	0	0	ARGs1
APH(6)	94,473	223,881	182,193	153,914	136,481	193,841	234,707	226,270	ARGs2
APH(4)	3.05×10^6	2.08×10^6	1.03×10^6	3.01×10^6	825,821	823,754	1.28×10^6	1.59×10^6	ARGs3
aadA16	60,323	123,913	119,410	54,093	69,271	22,020	597,774	153,366	ARGs4
AAC(6)	0	17,442	0	0	0	17,094	8,472	20,024	ARGs5
aacA_aphD	0	0	0	0	0	0	0	7,488	ARGs6
AAC(3)_Via	15,863	62.46×10^6	75.99×10^6	5.03×10^6	12,897	4.71×10^6	51.69×10^6	63.04×10^6	ARGs7
APH(6)_Id	0	24,544	897,374	50,248	40,056	24,369	235,464	436,311	ARGs8
aadA9	0	0	0	0	0	0	0	23,413	ARGs9
AAC(6)_Ir	17,868	66,399	18,288	19,684	0	0	27,720	39,156	ARGs10
aadA17	257,450	177,248	1.20×10^6	251,870	261,232	64,517	2.51×10^6	709,361	ARGs11
AAC(6)_IIa	12,267	0	109,816	17,574	0	0	106,013	13,582	ARGs12
aacA43	0	19,844	0	0	0	6,202	44,071	25,019	ARGs13
AAC(3)_Iic	0	0	0	0	0	0	0	32,185	ARGs14
AAC(6)_Iw	0	37,242	0	0	0	8,321	0	0	ARGs15
aadA21	29,869	37,863	374,730	56,012	66,814	69,248	419,248	116,005	ARGs16
APH(3)_Ia	0	0	19,615	0	15,511	15,531	0	170,687	ARGs17

APH3_III	0	0	0	0	0	0	66,930	17,270	ARGs18
AAC(3)_Xa	0	0	58,412	0	0	0	10,675	13,259	ARGs19
Aac(3)_iid_iia_iie	65,738	183,327	338,460	114,418	70,176	52,140	150,987	210,818	ARGs20
ANT(4)	0	0	0	0	0	0	0	6,454	ARGs21
APH(9)_Ib	0	0	26,270	0	0	15,085	20,612	40,407	ARGs22
armA	0	0	0	0	0	0	0	0	ARGs23
APH(6)_Ic	409,804	675,751	658,281	429,845	329,217	392,958	578,768	749,819	ARGs24
ANT(2)_Ia	0	20,022	40,371	0	0	0	0	0	ARGs25
aadA7	40,480	25,158	0	15,969	14,544	9,264	22,518	12,680	ARGs26
aadA	304,513	169,884	1.95×10^6	219,794	349,060	126,100	2.34×10^6	650,304	ARGs27
AAC(6)_Ib	0	16,000	75,402	0	82,565	118,096	1.04×10^6	333,276	ARGs28
aadA2	631,279	485,298	3.36×10^6	625,395	811,761	478,965	4.06×10^6	1.36×10^6	ARGs29
AAC(3)_Ib	0	0	0	0	0	6,262	13,088	7,628	ARGs30
AAC(3)_IV	176,688	239,286	300,687	134,445	361,948	392,829	226,855	358,949	ARGs31
APH(3)_Ia	0	0	0	0	0	0	5,563	49,008	ARGs32
APHA3	0	0	0	0	0	0	43,885	0	ARGs33
BEL beta_lac	18,911	9,589	0	13,065	0	0	0	7,611	ARGs34
blaSFO	74,942	1.10×10^6	83,413	16,708	10.66×10^6	7.57×10^6	1.56×10^6	9.91×10^6	ARGs35
CcrA	0	0	0	0	18,407	0	0	0	ARGs36
CARB beta_lac	0	6,542	0	0	0	22,821	14,102	25,933	ARGs37
IMIR beta_lac	0	0	0	0	0	0	0	0	ARGs38
CphA beta_lac	57,062	146,135	167,947	168,984	61,474	27,676	895,813	268,904	ARGs39
class C beta_lac	0	0	0	0	0	0	0	69,444	ARGs40
CARB_2	0	15,356	21,499	0	0	0	21,198	39,552	ARGs41
CTX_M_1_3_15	0	0	13,758	0	0	0	0	0	ARGs42
GES beta_lac	0	0	0	28,981	0	0	0	0	ARGs43
ACT beta_lac	14,666	25,462	0	0	7,059	0	7,910	0	ARGs44
MIR beta_lac	0	9,480	8,102	0	0	0	12,196	0	ARGs45
VIM beta_lac	0	0	0	8,851	7,339	0	6,257	0	ARGs46
Bla1	0	0	0	0	9,177	0	0	0	ARGs47
penA	49,508	0	0	39,110	0	0	0	0	ARGs48
OCH beta_lac	0	11,633	0	0	0	30,277	0	7,965	ARGs49
cphA2	0	0	0	0	0	0	0	0	ARGs50
VEB beta_lac	35,177	22,708	0	12,006	0	0	17,611	0	ARGs51
OXY_1_1	117,671	208,047	127,383	70,022	134,176	99,193	207,996	189,824	ARGs52
dfrA17	0	9,505	47,075	29,578	212,637	169,932	701,193	376,813	ARGs53
dfrA12	0	10,296	25,149	0	0	0	32,784	8,700	ARGs54
dfrB4	0	0	0	0	0	0	89,054	0	ARGs55
dfrA22	0	0	0	0	18,550	0	0	0	ARGs56
dfrA15	7,567	7,798	0	0	0	0	0	0	ARGs57
dfrA14	0	7,581	32,504	0	6,367	6,247	195,906	115,946	ARGs58
dfrA1	10,275	32,050	9,012	9,336	64,036	90,824	263,081	175,156	ARGs59
dfrA21	0	0	0	0	5,491	0	0	0	ARGs60
dfrA27	0	0	0	0	23,136	0	0	22,997	ARGs61
dfrK	0	0	11,945	0	0	0	0	8,183	ARGs62
QnrB4	785,871	2.65×10^6	897,217	736,483	642,850	607,762	1.71×10^6	1.01×10^6	ARGs63
qacA_B	0	0	0	0	0	0	7,436	5,744	ARGs64
QnrB_bob_resign	0	0	0	7,739	6,291	5,464	0	0	ARGs65
Qnrvc1_vc3_vc6	82,859	10,122	0	84,135	9,167	6,794	0	0	ARGs66
QnrS2	24,538	0	0	9,086	5,542	6,255	23,994	0	ARGs67

QnrB46_47_48	0	0	56,876	0	0	23,592	8,665	38,457	ARGs68
QnrS1_S3_S5	27,802	0	27,971	0	0	0	7,606	0	ARGs69
QepA_1_2	68,041	7,934	0	34,061	120,720	51,981	27,927	0	ARGs70
QnrD	0	26,444	0	0	0	0	22,127	20,572	ARGs71
FosX	0	0	11,013	0	0	0	0	0	ARGs72
vanA	21,755	34,916	37,116	0	9,118	0	5,839	5,832	ARGs73
vanHB	0	29,809	0	0	17,884	4,551	22,436	0	ARGs74
vanRB	0	0	0	0	6,360	0	0	19,860	ARGs75
vanSC	0	0	0	0	0	0	5,781	0	ARGs76
vanYD	0	28,053	0	0	0	0	0	0	ARGs77
vanB	20,687	111,634	66,388	44,683	18,951	5,184	60,353	64,978	ARGs78
ErmX	0	0	22,531	0	0	0	0	0	ARGs79
pikR2	12,336	67,305	33,815	7,953	12,254	38,381	59,495	7,178	ARGs80
lnuF	389,071	586,568	193,417	524,178	192,802	52,915	550,627	284,930	ARGs81
mef(B)	0	100,115	62,127	0	0	0	953,535	211,481	ARGs82
ErmQ	0	0	51,077	0	0	0	19,249	58,050	ARGs83
EreA	0	520,432	1.04×10^6	236,985	0	214,738	7.53×10^6	1.32×10^6	ARGs84
ErmE	165,083	13,350	0	94,309	131,183	75,706	42,257	0	ARGs85
ErmD	28,797	0	15,343	0	0	0	11,028	25,025	ARGs86
ErmF	0	9,300	0	0	88,824	53,210	434,841	23,404	ARGs87
EreB	0	0	320,231	15,430	15,012	176,592	5.87×10^6	427,557	ARGs88
ErmS	106,232	201,301	222,668	89,645	110,816	169,310	127,931	264,472	ARGs89
lnuC	0	32,292	9,562	0	9,104	0	2.14×10^6	220,271	ARGs90
erm(O)	146,056	0	0	30,739	6,575	0	0	0	ARGs91
oleC	74,978	125,937	54,976	50,790	45,950	80,050	42,987	134,921	ARGs92
vatB	0	22,621	0	0	0	0	0	7,668	ARGs93
vatA	62,354	0	0	29,757	21,613	39,486	0	22,261	ARGs94
ErmB	0	25,548	0	0	0	0	0	0	ARGs95
lnuB	0	6,734	0	0	0	0	75,104	59,495	ARGs96
pica	124,105	182,919	269,355	79,358	58,848	69,055	130,574	137,180	ARGs97
vgaB	0	0	0	0	0	14,943	0	0	ARGs98
mel_1	0	29,407	33,135	0	0	35,249	26,922	82,907	ARGs99
ttgB	350,525	143,703	140,991	139,180	254,810	194,986	220,591	231,546	ARGs100
optrA	0	16,443	0	0	0	8,758	0	7,584	ARGs101
OprD	214,883	417,307	342,568	246,726	154,176	131,482	297,851	273,570	ARGs102
multidrug resistance	0	0	13,601	0	0	0	0	0	ARGs103
merA_marko	1.11×10^6	88,483	154,613	743,736	344,770	402,937	222,944	606,571	ARGs104
ttgA	0	271,097	250,371	10,581	0	7,529	0	9,698	ARGs105
arsA	0	18,739	23,691	8,011	13,108	0	61,453	22,821	ARGs106
AcrF	0	19,967	237,574	6,389	0	18,505	232,217	98,086	ARGs107
czcA	0	1.75×10^6	1.92×10^6	468,228	0	496,148	1.28×10^6	3.75×10^6	ARGs108
MexE	0	93,675	179,404	0	10,815	11,583	0	0	ARGs109
nimE	19,249	0	0	0	0	0	0	0	ARGs110
mepA	76,102	160,774	35,886	49,519	89,659	84,121	104,061	35,655	ARGs111
copA	5.38×10^6	11.51×10^6	2.78×10^6	5.56×10^6	3.94×10^6	3.24×10^6	4.36×10^6	4.83×10^6	ARGs112
qacF_H	3.02×10^6	221,090	0	938,943	4.33×10^6	1.77×10^6	922,464	0	ARGs113
mdtG	223,436	550,522	548,766	218,787	301,644	300,540	459,294	441,892	ARGs114
ceoA	0	0	72,079	0	0	0	26,824	46,754	ARGs115
acrA	0	0	0	0	0	0	0	24,291	ARGs116

vgaA	0	14,763	0	0	0	0	0	0	ARGs117
tolC	0	0	0	0	0	0	0	6,646	ARGs118
MexB	0	47,901	0	0	0	0	6,330	9,384	ARGs119
acrR	0	0	0	0	0	0	6,760	0	ARGs120
terW	0	0	15,096	0	0	46,250	33,955	0	ARGs121
msrE	0	0	0	0	452,785	0	0	0	ARGs122
cefa_qacelta	0	1.35×10^6	3.22×10^6	142,899	0	193,567	1.48×10^6	1.12×10^6	ARGs123
pcoA	0	0	0	0	12,190	0	0	38,050	ARGs124
sugE	61,297	125,520	101,690	126,570	115,678	154,677	193,810	224,628	ARGs125
acrB	0	0	0	0	0	0	0	28,896	ARGs126
qacH_351	4.90×10^6	4.72×10^6	862,561	3.70×10^6	4.63×10^6	3.04×10^6	5.83×10^6	1.08×10^6	ARGs127
SAT_4	0	40,187	28,732	0	0	0	96,294	44,562	ARGs128
MCR_1.1	64,202	54,117	36,851	67,854	28,104	119,412	173,361	135,856	ARGs129
bacA	275,906	519,852	990,299	229,021	223,053	212,811	486,157	367,056	ARGs130
cmlv	169,218	432,069	447,038	398,215	273,610	400,881	486,935	450,242	ARGs131
catB9	23,531	30,896	0	0	0	10,654	11,536	0	ARGs132
catIII	0	0	0	0	7,831	0	0	0	ARGs133
cmx	13,752	23,531	121,921	0	0	84,125	65,666	379,341	ARGs134
cmlA1	42,282	27,287	386,216	102,078	100,197	323,444	43,766	72,164	ARGs135
catB3	10,341	0	0	0	0	10,255	50,104	0	ARGs136
cmlA5	45,214	0	325,902	96,208	181,581	44,868	21,921	26,436	ARGs137
catB8	0	0	0	0	0	16,430	10,670	0	ARGs138
floR	0	266,924	146,032	23,021	0	16,821	152,876	1.17×10^6	ARGs139
arr_2	0	10,671	0	9,366	0	0	25,350	53,641	ARGs140
arr_3	0	0	0	0	0	0	0	17,980	ARGs141
sulA_folP	0	0	0	24,159	0	12,244	0	12,679	ARGs142
sul1	4.91×10^6	11.51×10^6	18.71×10^6	6.60×10^6	6.65×10^6	6.09×10^6	12.15×10^6	14.42×10^6	ARGs143
sul2	0	0	76,510	0	0	0	7,936	123,389	ARGs144
tetO	0	28,474	44,473	0	6,441	17,389	191,372	62,623	ARGs145
tet36	0	0	14,214	14,684	0	13,895	0	617,438	ARGs146
tetJ	0	0	0	13,643	28,769	15,929	4,767	16,159	ARGs147
tetL	0	0	0	0	0	0	14,958	22,934	ARGs148
tetC	0	0	0	0	0	6,588	0	0	ARGs149
tetW	0	0	0	0	0	0	0	19,519	ARGs150
tetB	0	13,985	12,082	0	0	0	0	0	ARGs151
tetE	38,018	93,847	226,093	58,110	0	11,554	570,456	44,052	ARGs152
tet(44)	31,807	0	33,258	67,333	31,889	0	386,787	135,163	ARGs153
tetQ	6,607	0	0	0	0	0	64,563	699,493	ARGs154
tetT	0	0	0	0	0	0	8,106	0	ARGs155
tetB(P)	15,827	71,498	18,690	26,451	241,329	158,674	10.03×10^6	979,872	ARGs156
tetG	441,366	715,442	2.09×10^6	414,988	528,631	467,781	766,704	1.43×10^6	ARGs157
tetA	275,068	269,260	1.45×10^6	152,017	172,606	57,925	1.52×10^6	1.38×10^6	ARGs158
tetX	0	25,599	213,501	0	12,480	10,184	79,777	881,073	ARGs159
tetS	0	0	10,316	0	0	0	0	0	ARGs160
tetM	0	0	14,509	0	4,991	20,953	951,365	229,007	ARGs161
tetA(P)	540,126	797,272	613,603	548,510	754,964	926,058	971,954	1.11×10^6	ARGs162
tet32	54,163	80,436	279,306	0	84,169	52,524	958,296	281,504	ARGs163
tetR	331,161	63,276	1.21×10^6	0	0	25,607	827,485	865,186	ARGs164
tetD	395,487	364,450	660,197	376,968	202,760	175,085	330,495	266,704	ARGs165

Table S3D. ARG subtype -level abundance comparisons between RAS and PFRS

Feature	RAS mean ± SD	PFRS mean ± SD	Fold	P	BH q	Direction
tetA(P)	941,474 ± 147,588	624,878 ± 119,530	1.51	0.017	0.670	RAS > PFRS
blaSFO	$7.42 \times 10^6 \pm 4.12 \times 10^6$	319,936 ± 524,003	23.21	0.040	0.670	RAS > PFRS
tetD	243,761 ± 69,394	449,275 ± 141,191	0.54	0.054	0.670	PFRS > RAS
AAC(3)_IV	335,145 ± 73,800	212,776 ± 72,731	1.58	0.056	0.670	RAS > PFRS
sugE	172,198 ± 47,320	103,769 ± 30,557	1.66	0.058	0.670	RAS > PFRS
dfrA1	148,274 ± 89,999	15,168 ± 11,267	9.78	0.058	0.670	RAS > PFRS
dfrA17	365,143 ± 241,130	21,539 ± 21,020	16.95	0.065	0.670	RAS > PFRS
tetJ	16,406 ± 9,808	3,411 ± 6,821	4.81	0.078	0.670	RAS > PFRS
APH(4)	1.13×10^6 ± 372,102	2.29×10^6 ± 952,806	0.49	0.087	0.670	PFRS > RAS
AAC(3)_Ib	6,744 ± 5,377	0.00 ± 0.00	NA	0.087	0.670	RAS > PFRS
CARB beta_lac	15,714 ± 11,611	1,635 ± 3,271	9.61	0.090	0.670	RAS > PFRS
vanA	5,197 ± 3,795	23,447 ± 17,039	0.22	0.119	0.670	PFRS > RAS
BEL beta_lac	1,903 ± 3,806	10,391 ± 7,924	0.18	0.120	0.670	PFRS > RAS
MCR_1.1	114,183 ± 61,667	55,756 ± 13,878	2.05	0.153	0.670	RAS > PFRS
Qnrvc1_vc3_vc6	3,990 ± 4,708	44,279 ± 45,476	0.09	0.174	0.670	PFRS > RAS
ttgA	4,307 ± 5,051	133,012 ± 147,786	0.03	0.180	0.670	PFRS > RAS
dfrA27	11,533 ± 13,317	0.00 ± 0.00	NA	0.182	0.670	RAS > PFRS
dfrA15	0.00 ± 0.00	3,841 ± 4,437	0.00	0.182	0.670	PFRS > RAS
tetB	0.00 ± 0.00	6,517 ± 7,565	0.00	0.183	0.670	PFRS > RAS
penA	0.00 ± 0.00	22,154 ± 25,932	0.00	0.186	0.670	PFRS > RAS
qacA_B	3,295 ± 3,867	0.00 ± 0.00	NA	0.187	0.670	RAS > PFRS
OprD	214,270 ± 83,601	305,371 ± 92,269	0.70	0.194	0.670	PFRS > RAS
AAC(6)_Ib	393,829 ± 445,683	22,850 ± 35,837	17.24	0.194	0.670	RAS > PFRS
VEB beta_lac	4,403 ± 8,805	17,473 ± 15,011	0.25	0.195	0.670	PFRS > RAS
tetL	9,473 ± 11,413	0.00 ± 0.00	NA	0.195	0.670	RAS > PFRS
catB8	6,775 ± 8,169	0.00 ± 0.00	NA	0.196	0.670	RAS > PFRS
lnuB	33,650 ± 39,374	1,684 ± 3,367	19.99	0.203	0.670	RAS > PFRS
ANT(2)_Ia	0.00 ± 0.00	15,098 ± 19,312	0.00	0.216	0.670	PFRS > RAS
ErmF	150,070 ± 191,722	2,325 ± 4,650	64.55	0.221	0.670	RAS > PFRS
dfrA14	81,117 ± 92,345	10,021 ± 15,409	8.09	0.221	0.670	RAS > PFRS
pica	98,914 ± 40,675	163,934 ± 82,084	0.60	0.223	0.670	PFRS > RAS
QnrS1_S3_S5	1,902 ± 3,803	13,943 ± 16,100	0.14	0.233	0.670	PFRS > RAS
MexE	5,599 ± 6,473	68,270 ± 86,251	0.08	0.242	0.670	PFRS > RAS
pcoA	12,560 ± 17,939	0.00 ± 0.00	NA	0.256	0.670	RAS > PFRS
vanRB	6,555 ± 9,363	0.00 ± 0.00	NA	0.256	0.670	RAS > PFRS
terW	20,051 ± 23,691	3,774 ± 7,548	5.31	0.268	0.670	RAS > PFRS
tetM	301,579 ± 445,048	3,627 ± 7,254	83.15	0.273	0.670	RAS > PFRS
aacA43	18,823 ± 19,912	4,961 ± 9,922	3.79	0.275	0.670	RAS > PFRS
APH3_III	21,050 ± 31,652	0.00 ± 0.00	NA	0.276	0.670	RAS > PFRS
APH(9)_Ib	19,026 ± 16,705	6,568 ± 13,135	2.90	0.288	0.670	RAS > PFRS
AAC(6)	11,397 ± 9,043	4,360 ± 8,721	2.61	0.305	0.670	RAS > PFRS
lnuF	270,319 ± 209,809	423,308 ± 174,022	0.64	0.306	0.670	PFRS > RAS
erm(O)	1,644 ± 3,288	44,199 ± 69,434	0.04	0.308	0.670	PFRS > RAS
tet(44)	138,460 ± 175,315	33,100 ± 27,503	4.18	0.317	0.670	RAS > PFRS
tetO	69,456 ± 84,837	18,237 ± 22,048	3.81	0.318	0.670	RAS > PFRS
copA	4.09×10^6 ± 673,615	$6.31 \times 10^6 \pm 3.69 \times 10^6$	0.65	0.318	0.670	PFRS > RAS
tetB(P)	$2.85 \times 10^6 \pm 4.80 \times 10^6$	33,116 ± 25,978	86.16	0.325	0.670	RAS > PFRS

APH(3)_Ia	13,643 ± 23,722	0.00 ± 0.00	NA	0.333	0.670	RAS > PFRS
arr_2	19,748 ± 25,561	5,009 ± 5,809	3.94	0.336	0.670	RAS > PFRS
APH(3)_Ia	50,432 ± 80,503	4,904 ± 9,807	10.28	0.341	0.670	RAS > PFRS
tet32	344,123 ± 421,796	103,476 ± 121,910	3.33	0.343	0.670	RAS > PFRS
mel_1	36,269 ± 34,539	15,635 ± 18,118	2.32	0.343	0.670	RAS > PFRS
lnuC	592,448 ± 1.04 x 10^6	10,464 ± 15,235	56.62	0.343	0.670	RAS > PFRS
tetQ	191,014 ± 340,350	1,652 ± 3,303	115.65	0.347	0.670	RAS > PFRS
mef(B)	291,254 ± 452,636	40,560 ± 49,336	7.18	0.350	0.670	RAS > PFRS
EreB	1.62 x 10^6 ± 2.84 x 10^6	83,915 ± 157,712	19.33	0.358	0.670	RAS > PFRS
cmx	132,283 ± 168,615	39,801 ± 55,591	3.32	0.362	0.670	RAS > PFRS
APH(6)	197,825 ± 44,528	163,615 ± 54,320	1.21	0.369	0.670	RAS > PFRS
catB3	15,090 ± 23,838	2,585 ± 5,171	5.84	0.375	0.670	RAS > PFRS
vanB	37,367 ± 29,808	60,848 ± 38,661	0.61	0.375	0.670	PFRS > RAS
EreA	2.27 x 10^6 ± 3.56 x 10^6	450,058 ± 448,800	5.04	0.384	0.670	RAS > PFRS
bacA	322,269 ± 129,987	503,769 ± 348,510	0.64	0.387	0.670	PFRS > RAS
AAC(3)_Iic	8,046 ± 16,092	0.00 ± 0.00	NA	0.391	0.670	RAS > PFRS
ANT(4)	1,614 ± 3,227	0.00 ± 0.00	NA	0.391	0.670	RAS > PFRS
APH(3)	0.00 ± 0.00	2,544 ± 5,087	0.00	0.391	0.670	PFRS > RAS
APHA3	10,971 ± 21,942	0.00 ± 0.00	NA	0.391	0.670	RAS > PFRS
Bla1	2,294 ± 4,589	0.00 ± 0.00	NA	0.391	0.670	RAS > PFRS
CTX_M_1_3_15	0.00 ± 0.00	3,440 ± 6,879	0.00	0.391	0.670	PFRS > RAS
CcrA	4,602 ± 9,204	0.00 ± 0.00	NA	0.391	0.670	RAS > PFRS
ErmB	0.00 ± 0.00	6,387 ± 12,774	0.00	0.391	0.670	PFRS > RAS
ErmX	0.00 ± 0.00	5,633 ± 11,265	0.00	0.391	0.670	PFRS > RAS
FosX	0.00 ± 0.00	2,753 ± 5,506	0.00	0.391	0.670	PFRS > RAS
GES beta_lac	0.00 ± 0.00	7,245 ± 14,490	0.00	0.391	0.670	PFRS > RAS
aacA_aphD	1,872 ± 3,744	0.00 ± 0.00	NA	0.391	0.670	RAS > PFRS
aadA9	5,853 ± 11,707	0.00 ± 0.00	NA	0.391	0.670	RAS > PFRS
acrA	6,073 ± 12,145	0.00 ± 0.00	NA	0.391	0.670	RAS > PFRS
acrB	7,224 ± 14,448	0.00 ± 0.00	NA	0.391	0.670	RAS > PFRS
acrR	1,690 ± 3,380	0.00 ± 0.00	NA	0.391	0.670	RAS > PFRS
arr_3	4,495 ± 8,990	0.00 ± 0.00	NA	0.391	0.670	RAS > PFRS
catIII	1,958 ± 3,916	0.00 ± 0.00	NA	0.391	0.670	RAS > PFRS
class C beta_lac	17,361 ± 34,722	0.00 ± 0.00	NA	0.391	0.670	RAS > PFRS
dfrA21	1,373 ± 2,745	0.00 ± 0.00	NA	0.391	0.670	RAS > PFRS
dfrA22	4,637 ± 9,275	0.00 ± 0.00	NA	0.391	0.670	RAS > PFRS
dfrB4	22,263 ± 44,527	0.00 ± 0.00	NA	0.391	0.670	RAS > PFRS
msrE	113,196 ± 226,393	0.00 ± 0.00	NA	0.391	0.670	RAS > PFRS
multidrug resistance	0.00 ± 0.00	3,400 ± 6,800	0.00	0.391	0.670	PFRS > RAS
nimE	0.00 ± 0.00	4,812 ± 9,625	0.00	0.391	0.670	PFRS > RAS
tetC	1,647 ± 3,294	0.00 ± 0.00	NA	0.391	0.670	RAS > PFRS
tetS	0.00 ± 0.00	2,579 ± 5,158	0.00	0.391	0.670	PFRS > RAS
tetT	2,026 ± 4,053	0.00 ± 0.00	NA	0.391	0.670	RAS > PFRS
tetW	4,880 ± 9,759	0.00 ± 0.00	NA	0.391	0.670	RAS > PFRS
tolC	1,661 ± 3,323	0.00 ± 0.00	NA	0.391	0.670	RAS > PFRS
vanSC	1,445 ± 2,890	0.00 ± 0.00	NA	0.391	0.670	RAS > PFRS
vanYD	0.00 ± 0.00	7,013 ± 14,027	0.00	0.391	0.670	PFRS > RAS
vgaA	0.00 ± 0.00	3,691 ± 7,382	0.00	0.391	0.670	PFRS > RAS
vgaB	3,736 ± 7,472	0.00 ± 0.00	NA	0.391	0.670	RAS > PFRS

ACT beta_lac	3,742 ± 4,335	10,032 ± 12,394	0.37	0.396	0.670	PFRS > RAS
tet36	157,833 ± 306,473	7,225 ± 8,344	21.85	0.398	0.670	RAS > PFRS
catB9	5,547 ± 6,416	13,607 ± 15,997	0.41	0.403	0.672	PFRS > RAS
AAC(6)_Ir	16,719 ± 19,862	30,560 ± 23,906	0.55	0.409	0.674	PFRS > RAS
aadA16	210,608 ± 263,765	89,435 ± 37,345	2.35	0.428	0.699	RAS > PFRS
OCH beta_lac	9,560 ± 14,312	2,908 ± 5,816	3.29	0.438	0.708	RAS > PFRS
CphA beta_lac	313,467 ± 402,612	135,032 ± 53,037	2.32	0.442	0.708	RAS > PFRS
tetX	245,878 ± 424,691	59,775 ± 103,192	4.11	0.451	0.715	RAS > PFRS
arsA	24,346 ± 26,447	12,610 ± 10,654	1.93	0.457	0.718	RAS > PFRS
Aac(3)_iid_iia_iie	121,030 ± 73,691	175,486 ± 118,878	0.69	0.471	0.734	PFRS > RAS
floR	335,645 ± 562,341	108,994 ± 123,268	3.08	0.484	0.742	RAS > PFRS
QepA_1_2	50,157 ± 51,615	27,509 ± 30,690	1.82	0.485	0.742	RAS > PFRS
AAC(6)_Iw	2,080 ± 4,160	9,311 ± 18,621	0.22	0.499	0.755	PFRS > RAS
OXY_1_1	157,797 ± 50,127	130,781 ± 57,286	1.21	0.505	0.757	RAS > PFRS
SAT_4	35,214 ± 45,819	17,230 ± 20,438	2.04	0.512	0.761	RAS > PFRS
aadA17	887,275 ± 1.12 x 10^6	472,210 ± 488,079	1.88	0.533	0.785	RAS > PFRS
MexB	3,928 ± 4,704	11,975 ± 23,950	0.33	0.554	0.808	PFRS > RAS
qacF_H	1.76 x 10^6 ± 1.86 x 10^6	1.04 x 10^6 ± 1.38 x 10^6	1.68	0.564	0.808	RAS > PFRS
aadA7	14,752 ± 5,620	20,402 ± 16,947	0.72	0.564	0.808	PFRS > RAS
vatB	1,917 ± 3,834	5,655 ± 11,311	0.34	0.568	0.808	PFRS > RAS
cmlA5	68,701 ± 75,905	116,831 ± 144,815	0.59	0.584	0.819	PFRS > RAS
cefa_qacelta	698,372 ± 714,392	1.18 x 10^6 ± 1.49 x 10^6	0.59	0.590	0.819	PFRS > RAS
ttgB	225,483 ± 24,838	193,600 ± 104,634	1.16	0.591	0.819	RAS > PFRS
AAC(3)_Xa	5,984 ± 6,989	14,603 ± 29,206	0.41	0.602	0.828	PFRS > RAS
CARB_2	15,187 ± 19,071	9,214 ± 10,931	1.65	0.611	0.833	RAS > PFRS
cmlv	402,917 ± 93,137	361,635 ± 129,894	1.11	0.626	0.843	RAS > PFRS
QnrB4	993,348 ± 511,277	1.27 x 10^6 ± 922,046	0.78	0.628	0.843	PFRS > RAS
tetA	783,328 ± 775,078	536,749 ± 611,899	1.46	0.636	0.847	RAS > PFRS
merA_marko	394,306 ± 160,156	524,636 ± 489,833	0.75	0.642	0.848	PFRS > RAS
QnrD	10,675 ± 12,342	6,611 ± 13,222	1.61	0.669	0.876	RAS > PFRS
vanHB	11,218 ± 10,655	7,452 ± 14,904	1.51	0.697	0.902	RAS > PFRS
VIM beta_lac	3,399 ± 3,950	2,213 ± 4,426	1.54	0.703	0.902	RAS > PFRS
QnrB_bob_resign	2,939 ± 3,410	1,935 ± 3,869	1.52	0.711	0.902	RAS > PFRS
sul2	32,831 ± 60,487	19,127 ± 38,255	1.72	0.717	0.902	RAS > PFRS
aadA2	1.68 x 10^6 ± 1.63 x 10^6	1.27 x 10^6 ± 1.39 x 10^6	1.32	0.719	0.902	RAS > PFRS
aadA21	167,829 ± 169,135	124,619 ± 167,099	1.35	0.729	0.902	RAS > PFRS
czcA	1.38 x 10^6 ± 1.66 x 10^6	1.03 x 10^6 ± 944,946	1.34	0.732	0.902	RAS > PFRS
tetE	156,515 ± 276,590	104,017 ± 84,596	1.50	0.737	0.902	RAS > PFRS
ErmQ	19,325 ± 27,365	12,769 ± 25,539	1.51	0.738	0.902	RAS > PFRS
MIR beta_lac	3,049 ± 6,098	4,395 ± 5,107	0.69	0.747	0.906	PFRS > RAS
aadA	867,421 ± 1.01 x 10^6	659,818 ± 858,642	1.31	0.765	0.921	RAS > PFRS
ErmS	168,132 ± 68,760	154,962 ± 66,764	1.08	0.793	0.938	RAS > PFRS
AcrF	87,202 ± 105,627	65,982 ± 114,697	1.32	0.795	0.938	RAS > PFRS
dfrK	2,046 ± 4,092	2,986 ± 5,972	0.69	0.805	0.938	PFRS > RAS
APH(6)_Ic	512,690 ± 190,258	543,420 ± 143,128	0.94	0.806	0.938	PFRS > RAS
tetG	798,330 ± 440,555	915,848 ± 795,518	0.87	0.807	0.938	PFRS > RAS
APH(6)_Id	184,050 ± 193,659	243,042 ± 436,704	0.76	0.817	0.940	PFRS > RAS
AAC(3)_Via	29.86 x 10^6 ± 32.15 x 10^6	35.87 x 10^6 ± 38.96 x 10^6	0.83	0.820	0.940	PFRS > RAS
ErmD	9,013 ± 11,873	11,035 ± 13,876	0.82	0.832	0.947	PFRS > RAS
QnrB46_47_48	17,679 ± 16,936	14,219 ± 28,438	1.24	0.843	0.953	RAS > PFRS

sul1	9.83 x 10^{6} ± 4.11 x 10^{6}	10.43 x 10^{6} ± 6.19 x 10^{6}	0.94	0.877	0.984	PFRS > RAS
dfrA12	10,371 ± 15,494	8,861 ± 11,894	1.17	0.883	0.984	RAS > PFRS
AAC(6)_IIa	29,899 ± 51,145	34,914 ± 50,474	0.86	0.894	0.989	PFRS > RAS
vatA	20,840 ± 16,172	23,028 ± 29,734	0.91	0.903	0.989	PFRS > RAS
ErmE	62,287 ± 55,400	68,185 ± 76,872	0.91	0.905	0.989	PFRS > RAS
mdtG	375,843 ± 86,608	385,378 ± 189,689	0.98	0.931	1.000	PFRS > RAS
tetR	429,570 ± 481,600	401,648 ± 559,078	1.07	0.942	1.000	RAS > PFRS
qacH_351	3.65 x 10^{6} ± 2.06 x 10^{6}	3.55 x 10^{6} ± 1.87 x 10^{6}	1.03	0.946	1.000	RAS > PFRS
QnrS2	8,948 ± 10,413	8,406 ± 11,576	1.06	0.947	1.000	RAS > PFRS
mepA	78,374 ± 29,694	80,570 ± 56,016	0.97	0.948	1.000	PFRS > RAS
pikR2	29,327 ± 24,318	30,352 ± 27,103	0.97	0.957	1.000	PFRS > RAS
cmlA1	134,893 ± 127,795	139,466 ± 167,643	0.97	0.967	1.000	PFRS > RAS
sulA_folP	6,231 ± 7,197	6,040 ± 12,079	1.03	0.979	1.000	RAS > PFRS
oleC	75,977 ± 42,743	76,670 ± 34,499	0.99	0.981	1.000	PFRS > RAS
ceoA	18,394 ± 22,745	18,020 ± 36,039	1.02	0.987	1.000	RAS > PFRS
optrA	4,085 ± 4,742	4,111 ± 8,221	0.99	0.996	1.000	PFRS > RAS
IMIR beta_lac	0.00 ± 0.00	0.00 ± 0.00	NA	1.000	1.000	Equal
armA	0.00 ± 0.00	0.00 ± 0.00	NA	1.000	1.000	Equal
cphA2	0.00 ± 0.00	0.00 ± 0.00	NA	1.000	1.000	Equal

Table S4A. Total MGE class abundance comparison between RAS and PFRS

Metric	RAS mean ± SD	PFRS mean ± SD	RAS/PFRS fold	P value
MGE class total abundance	38.90 x 10^{6} ± 23.18 x 10^{6}	29.41 x 10^{6} ± 13.02 x 10^{6}	1.32	0.509

Table S4B. MGE class-level abundance comparison between RAS and PFRS

Feature	RAS mean ± SD	PFRS mean ± SD	Fold	P	BH q	Direction
IS	567,216 ± 142,341	1.12 x 10^{6} ± 1.57 x 10^{6}	0.50	0.529	0.806	PFRS > RAS
In	9.22 x 10^{6} ± 3.64 x 10^{6}	10.17 x 10^{6} ± 6.35 x 10^{6}	0.91	0.806	0.806	PFRS > RAS
Tn	8.02 x 10^{6} ± 10.62 x 10^{6}	2.46 x 10^{6} ± 557,435	3.26	0.372	0.806	RAS > PFRS
ICE	9.39 x 10^{6} ± 8.98 x 10^{6}	2.74 x 10^{6} ± 2.28 x 10^{6}	3.43	0.237	0.806	RAS > PFRS
Plasmid	11.70 x 10^{6} ± 3.69 x 10^{6}	12.92 x 10^{6} ± 3.71 x 10^{6}	0.91	0.659	0.806	PFRS > RAS

Table S4C. MGE subtype/gene abundance comparison between RAS and PFRS

Feature	RAS mean ± SD	PFRS mean ± SD	Fold	P	BH q	Direction
orf37-IS26	4,421 ± 8,842	10,878 ± 7,773	0.41	0.315	0.733	PFRS > RAS
ISSm2-Xanthob	434,655 ± 88,284	991,929 ± 1.56 x 10^{6}	0.44	0.527	0.733	PFRS > RAS
IS1133	26,489 ± 38,293	8,381 ± 11,141	3.16	0.422	0.733	RAS > PFRS
ISPps1-pseud	91,524 ± 87,188	111,403 ± 15,989	0.82	0.682	0.859	PFRS > RAS
orf39-IS26	10,127 ± 11,783	2,230 ± 4,461	4.54	0.281	0.733	RAS > PFRS
intl3	3,309 ± 6,618	39,497 ± 29,411	0.08	0.088	0.733	PFRS > RAS
intl2	3.31 x 10^{6} ± 1.38 x 10^{6}	3.57 x 10^{6} ± 2.17 x 10^{6}	0.93	0.846	0.904	PFRS > RAS
intI1	5.92 x 10^{6} ± 2.27 x 10^{6}	6.57 x 10^{6} ± 4.16 x 10^{6}	0.90	0.795	0.904	PFRS > RAS
Tn3	36,485 ± 47,327	126,796 ± 244,415	0.29	0.517	0.733	PFRS > RAS

IS21-ISAs29	126,595 ± 226,580	104,124 ± 162,111	1.22	0.878	0.904	RAS > PFRS
IS256	26,603 ± 53,206	6,405 ± 12,810	4.15	0.509	0.733	RAS > PFRS
IS6100	7.92 x 10^6 ± 7.58 x 10^6	2.18 x 10^6 ± 1.74 x 10^6	3.63	0.228	0.733	RAS > PFRS
lncF_FIC	1,411 ± 2,822	0.00 ± 0.00		0.391	0.733	RAS > PFRS
IS630	115,787 ± 123,302	105,845 ± 131,871	1.09	0.916	0.916	RAS > PFRS
mobA	1,811 ± 3,622	8,651 ± 10,597	0.21	0.294	0.733	PFRS > RAS
ISEcp1	781,306 ± 1.38 x 10^6	20,564 ± 16,152	37.99	0.349	0.733	RAS > PFRS
ISCR1	12,825 ± 12,109	20,538 ± 9,813	0.62	0.362	0.733	PFRS > RAS
IS1247	76,445 ± 62,514	38,844 ± 58,795	1.97	0.415	0.733	RAS > PFRS
IncI1_repI1	132,696 ± 49,350	61,766 ± 24,714	2.15	0.056	0.733	RAS > PFRS
cro	5,889 ± 7,572	6,906 ± 9,992	0.85	0.877	0.904	PFRS > RAS
IS26	155,165 ± 243,478	56,935 ± 113,870	2.73	0.503	0.733	RAS > PFRS
trb-C	11.30 x 10^6 ± 3.42 x 10^6	12.63 x 10^6 ± 3.59 x 10^6	0.89	0.612	0.800	PFRS > RAS
IncP_oriT	18,940 ± 23,330	0.00 ± 0.00		0.203	0.733	RAS > PFRS
IncN_rep	15,221 ± 16,235	2,238 ± 4,476	6.80	0.209	0.733	RAS > PFRS
pBS228-IncP	369,205 ± 345,582	288,511 ± 404,750	1.28	0.772	0.904	RAS > PFRS
trfa	0.00 ± 0.00	48,353 ± 74,522	0.00	0.285	0.733	PFRS > RAS
tnpA-3	55,681 ± 54,963	47,138 ± 34,115	1.18	0.802	0.904	RAS > PFRS
tnpA-6	15,982 ± 14,906	4,476 ± 8,952	3.57	0.244	0.733	RAS > PFRS
tnpA-1	839,560 ± 914,081	429,123 ± 293,993	1.96	0.446	0.733	RAS > PFRS
tnpA-5	2.73 x 10^6 ± 5.06 x 10^6	81,257 ± 70,270	33.56	0.373	0.733	RAS > PFRS
tnpA-2	4.12 x 10^6 ± 4.51 x 10^6	1.15 x 10^6 ± 775,741	3.59	0.280	0.733	RAS > PFRS
tnpA-4	175,096 ± 92,034	477,156 ± 870,756	0.37	0.539	0.733	PFRS > RAS
IS613	86,256 ± 72,968	225,770 ± 317,188	0.38	0.449	0.733	PFRS > RAS
tnpA-7	3,441 ± 6,882	0.00 ± 0.00		0.391	0.733	RAS > PFRS

Table S5A. Co-occurrence network topology and modularity statistics

Parameter	Value
Number of nodes	60
Number of edges	96
Positive edges	65
Negative edges	31
Network density	0.0542
Average degree	3.20
Modularity algorithm	Louvain community detection
Modularity value (Q)	0.4792
Number of modules	10

Table S5B. Significant co-occurrence associations among microbial species, ARGs, and MGEs

Source node	Target node	Source category	Target category	Spearman's ρ	P value	FDR-adjusted q value	Correlation type
g__Flavobacterium	tetB(P)	Species	ARG	-0.8144	0.0138	0.0339	Negative
g__Rhodoluna	sul1	Species	ARG	-0.7306	0.0396	0.0442	Negative
g__norank_f__Steroidobacteraceae	tetB(P)	Species	ARG	0.7665	0.0265	0.0398	Positive
g__norank_f__Steroidobacteraceae	tnpA-2	Species	MGE	0.8383	0.0093	0.0339	Positive
g__Exiguobacterium	tetB(P)	Species	ARG	0.7904	0.0195	0.0372	Positive
g__Sphaerotilus	blaSFO	Species	ARG	-0.8225	0.0122	0.0339	Negative
g__Sphaerotilus	tetB(P)	Species	ARG	-0.8839	0.0036	0.0326	Negative
g__Bacillus	blaSFO	Species	ARG	0.7785	0.0229	0.0378	Positive
g__Bacillus	tetB(P)	Species	ARG	0.8503	0.0075	0.0326	Positive
g__Clostridium_sensu_stricto_1	copA	Species	ARG	-0.8333	0.0102	0.0339	Negative
g__Clostridium_sensu_stricto_1	QnrB4	Species	ARG	-0.7857	0.0208	0.0372	Negative
g__Clostridium_sensu_stricto_1	EreB	Species	ARG	0.8264	0.0114	0.0339	Positive
g__Clostridium_sensu_stricto_1	IS6100	Species	MGE	0.7619	0.0280	0.0398	Positive
g__Gemmobacter	blaSFO	Species	ARG	-0.7619	0.0280	0.0398	Negative
g__Gemmobacter	tetB(P)	Species	ARG	-0.7857	0.0208	0.0372	Negative
g__Gemmobacter	IS6100	Species	MGE	-0.7143	0.0465	0.0489	Negative
g__unclassified_f__Rhodobacteraceae	aadA2	Species	ARG	-0.7619	0.0280	0.0398	Negative
g__unclassified_f__Rhodobacteraceae	aadA	Species	ARG	-0.8095	0.0149	0.0339	Negative
g__unclassified_f__Rhodobacteraceae	aadA17	Species	ARG	-0.7143	0.0465	0.0489	Negative
g__unclassified_f__Rhodobacteraceae	tetA	Species	ARG	-0.7143	0.0465	0.0489	Negative
g__Sphingorhabdus	blaSFO	Species	ARG	-0.8571	0.0065	0.0326	Negative

g__Rhodoferax	blaSFO	Species	ARG	-0.8264	0.0114	0.0339	Negative
g__Rhodoferax	tetB(P)	Species	ARG	-0.7785	0.0229	0.0378	Negative
g__Clostridium_sensu_stricto_13	blaSFO	Species	ARG	0.7619	0.0280	0.0398	Positive
g__norank_f__SC-I-84	tetB(P)	Species	ARG	0.7306	0.0396	0.0442	Positive
g__norank_f__SC-I-84	tnpA-2	Species	MGE	0.7665	0.0265	0.0398	Positive
g__norank_f__norank_o__SJA-15	tetB(P)	Species	ARG	0.7545	0.0305	0.0401	Positive
g__norank_f__norank_o__SJA-15	EreB	Species	ARG	0.8313	0.0105	0.0339	Positive
g__norank_f__norank_o__Vicinamibacterales	tetB(P)	Species	ARG	0.9271	0.0009	0.0326	Positive
g__norank_f__norank_o__Vicinamibacterales	EreB	Species	ARG	0.7609	0.0283	0.0398	Positive
g__norank_f__Sutterellaceae	blaSFO	Species	ARG	0.7563	0.0299	0.0398	Positive
g__norank_f__Sutterellaceae	tetB(P)	Species	ARG	0.8539	0.0070	0.0326	Positive
g__norank_f__Sutterellaceae	tnpA-2	Species	MGE	0.7319	0.0390	0.0442	Positive
g__Candidatus_Competibacter	blaSFO	Species	ARG	0.7075	0.0496	0.0496	Positive
g__Candidatus_Competibacter	tetB(P)	Species	ARG	0.8051	0.0159	0.0339	Positive
g__Candidatus_Competibacter	EreB	Species	ARG	0.7363	0.0372	0.0436	Positive
g__norank_f__norank_o__SBR1031	tetB(P)	Species	ARG	0.8539	0.0070	0.0326	Positive
g__norank_f__norank_o__SBR1031	EreB	Species	ARG	0.7363	0.0372	0.0436	Positive
g__norank_f__Methylophilaceae	copA	Species	ARG	0.7619	0.0280	0.0398	Positive
g__norank_f__Methylophilaceae	QnrB4	Species	ARG	0.8810	0.0039	0.0326	Positive
g__norank_f__norank_o__PeM15	blaSFO	Species	ARG	0.7381	0.0366	0.0436	Positive
g__norank_f__norank_o__PeM15	tetB(P)	Species	ARG	0.8095	0.0149	0.0339	Positive
g__Hydrogenophaga	blaSFO	Species	ARG	-0.9157	0.0014	0.0326	Negative
g__Hydrogenophaga	tetB(P)	Species	ARG	-0.8314	0.0105	0.0339	Negative
g__Pseudorhodobacter	blaSFO	Species	ARG	-0.7857	0.0208	0.0372	Negative

g__Pseudorhodobacter	tetB(P)	Species	ARG	-0.8095	0.0149	0.0339	Negative
g__Pseudorhodobacter	IS6100	Species	MGE	-0.7381	0.0366	0.0436	Negative
g__hgcI_clade	copA	Species	ARG	0.8729	0.0047	0.0326	Positive
g__hgcI_clade	APH(4)	Species	ARG	0.7910	0.0194	0.0372	Positive
g__hgcI_clade	QnrB4	Species	ARG	0.7910	0.0194	0.0372	Positive
g__hgcI_clade	EreB	Species	ARG	-0.7135	0.0469	0.0489	Negative
g__hgcI_clade	APH(6)_Ic	Species	ARG	0.7092	0.0488	0.0496	Positive
g__hgcI_clade	IS6100	Species	MGE	-0.8729	0.0047	0.0326	Negative
g__norank_f__norank_o__norank_c__KD4-96	tetB(P)	Species	ARG	0.7785	0.0229	0.0378	Positive
g__norank_f__norank_o__norank_c__KD4-96	EreB	Species	ARG	0.8795	0.0040	0.0326	Positive
g__norank_f__Rhizobiales_Incertae_Sedis	tetB(P)	Species	ARG	0.7425	0.0349	0.0436	Positive
g__Parablastomonas	APH(6)_Ic	Species	ARG	0.7306	0.0396	0.0442	Positive
g__Parablastomonas	intI1	Species	MGE	0.8503	0.0075	0.0326	Positive
g__Parablastomonas	intl2	Species	MGE	0.8503	0.0075	0.0326	Positive
g__norank_f__Anaerolineaceae	tetB(P)	Species	ARG	0.8503	0.0075	0.0326	Positive
g__norank_f__Anaerolineaceae	EreB	Species	ARG	0.9036	0.0021	0.0326	Positive
g__norank_f__norank_o__Gaiellales	blaSFO	Species	ARG	0.7563	0.0299	0.0398	Positive
g__norank_f__norank_o__Gaiellales	tetB(P)	Species	ARG	0.7563	0.0299	0.0398	Positive
g__Aurantimicrobium	APH(4)	Species	ARG	0.7143	0.0465	0.0489	Positive
g__Aurantimicrobium	QnrB4	Species	ARG	0.7381	0.0366	0.0436	Positive
g__Paenisporosarcina	blaSFO	Species	ARG	0.8051	0.0159	0.0339	Positive
g__Paenisporosarcina	tetB(P)	Species	ARG	0.8051	0.0159	0.0339	Positive
g__Romboutsia	tetB(P)	Species	ARG	0.8675	0.0052	0.0326	Positive
g__Romboutsia	EreB	Species	ARG	0.7515	0.0316	0.0409	Positive

g__Romboutsia	IS6100	Species	MGE	0.8193	0.0128	0.0339	Positive
g__Dechloromonas	tnpA-2	Species	MGE	0.7381	0.0366	0.0436	Positive
g__Sporacetigenium	blaSFO	Species	ARG	0.8625	0.0058	0.0326	Positive
g__Sporacetigenium	tetB(P)	Species	ARG	0.7864	0.0206	0.0372	Positive
g__norank_f__norank_o__OPB41	tetB(P)	Species	ARG	0.8051	0.0159	0.0339	Positive
g__norank_f__norank_o__OPB41	EreB	Species	ARG	0.7609	0.0283	0.0398	Positive
g__Polynucleobacter	blaSFO	Species	ARG	-0.7807	0.0222	0.0378	Negative
g__Arenimonas	sul1	Species	ARG	0.7857	0.0208	0.0372	Positive
g__Trichococcus	copA	Species	ARG	-0.9027	0.0021	0.0326	Negative
g__Trichococcus	qacH_351	Species	ARG	-0.7075	0.0496	0.0496	Negative
g__Trichococcus	APH(4)	Species	ARG	-0.8051	0.0159	0.0339	Negative
g__Trichococcus	EreA	Species	ARG	0.7854	0.0209	0.0372	Positive
g__Trichococcus	QnrB4	Species	ARG	-0.8539	0.0070	0.0326	Negative
g__Trichococcus	EreB	Species	ARG	0.8590	0.0063	0.0326	Positive
g__Trichococcus	APH(6)_Ic	Species	ARG	-0.8051	0.0159	0.0339	Negative
g__Trichococcus	trb-C	Species	MGE	-0.8051	0.0159	0.0339	Negative
g__Trichococcus	IS6100	Species	MGE	0.9515	0.0003	0.0264	Positive
g__Novosphingobium	blaSFO	Species	ARG	-0.8571	0.0065	0.0326	Negative
g__Pseudomonas	aadA2	Species	ARG	0.7143	0.0465	0.0489	Positive
g__Pseudomonas	aadA	Species	ARG	0.7619	0.0280	0.0398	Positive
g__Pseudomonas	aadA17	Species	ARG	0.8333	0.0102	0.0339	Positive
g__Pseudomonas	tetA	Species	ARG	0.8095	0.0149	0.0339	Positive
g__Thiobacillus	blaSFO	Species	ARG	0.7075	0.0496	0.0496	Positive
g__Thiobacillus	tetB(P)	Species	ARG	0.8051	0.0159	0.0339	Positive

g__Thiobacillus	EreB	Species	ARG	0.7363	0.0372	0.0436	Positive
g__Ellin6067	tetB(P)	Species	ARG	0.8051	0.0159	0.0339	Positive
g__Ellin6067	tnpA-2	Species	MGE	0.7563	0.0299	0.0398	Positive

Table S5C. Network centrality metrics and module assignment of key nodes

Node ID	Node name	Node category	Module ID	Degree	Degree centrality	Betweenness centrality	Closeness centrality
2	tetB(P)	ARG	M1	26	0.4407	0.3588	0.3750
9	blaSFO	ARG	M2	17	0.2881	0.1612	0.3235
14	EreB	ARG	M1	12	0.2034	0.2270	0.3502
53	g__Trichococcus	Species	M6	9	0.1525	0.1754	0.3027
15	IS6100	MGE	M9	6	0.1017	0.0918	0.3186
35	g__hgcI_clade	Species	M5	6	0.1017	0.0913	0.2902
13	QnrB4	ARG	M5	5	0.0848	0.0325	0.2341
6	tnpA-2	MGE	M4	5	0.0848	0.0310	0.2242
11	g__Clostridium_sensu_stricto_1	Species	M5	4	0.0678	0.0264	0.2716
12	copA	ARG	M5	4	0.0678	0.0155	0.2316
17	g__unclassified_f__Rhodobacteraceae	Species	M3	4	0.0678	0.0018	0.0706
58	g__Pseudomonas	Species	M3	4	0.0678	0.0018	0.0706
37	APH(6)_Ic	ARG	M7	3	0.0509	0.0828	0.2341
40	g__Parablastomonas	Species	M7	3	0.0509	0.0567	0.1875
16	g__Gemmobacter	Species	M9	3	0.0509	0.0368	0.3531
34	g__Pseudorhodobacter	Species	M9	3	0.0509	0.0368	0.3531
29	g__Candidatus_Competibacter	Species	M1	3	0.0509	0.0350	0.3531
59	g__Thiobacillus	Species	M1	3	0.0509	0.0350	0.3531

47	g__Romboutsia	Species	M1	3	0.0509	0.0282	0.3310
28	g__norank_f__Sutterellaceae	Species	M4	3	0.0509	0.0243	0.2863
36	APH(4)	ARG	M10	3	0.0509	0.0116	0.2290
26	g__norank_f__norank_o__SJA-15	Species	M1	2	0.0339	0.0125	0.3260
27	g__norank_f__norank_o__Vicinamibacterales	Species	M1	2	0.0339	0.0125	0.3260
30	g__norank_f__norank_o__SBR1031	Species	M1	2	0.0339	0.0125	0.3260
38	g__norank_f__norank_o__norank_c__KD4-96	Species	M1	2	0.0339	0.0125	0.3260
43	g__norank_f__Anaerolineaceae	Species	M1	2	0.0339	0.0125	0.3260
50	g__norank_f__norank_o__OPB41	Species	M1	2	0.0339	0.0125	0.3260
5	g__norank_f__Steroidobacteraceae	Species	M4	2	0.0339	0.0106	0.2682
25	g__norank_f__SC-I-84	Species	M4	2	0.0339	0.0106	0.2682
60	g__Ellin6067	Species	M4	2	0.0339	0.0106	0.2682
8	g__Sphaerotilus	Species	M2	2	0.0339	0.0029	0.2788
10	g__Bacillus	Species	M2	2	0.0339	0.0029	0.2788
23	g__Rhodoferax	Species	M2	2	0.0339	0.0029	0.2788
32	g__norank_f__norank_o__PeM15	Species	M2	2	0.0339	0.0029	0.2788
33	g__Hydrogenophaga	Species	M2	2	0.0339	0.0029	0.2788
44	g__norank_f__norank_o__Gaiellales	Species	M2	2	0.0339	0.0029	0.2788
46	g__Paenisporosarcina	Species	M2	2	0.0339	0.0029	0.2788
49	g__Sporacetigenium	Species	M2	2	0.0339	0.0029	0.2788
4	sul1	ARG	M8	2	0.0339	0.0006	0.0339
45	g__Aurantimicrobium	Species	M10	2	0.0339	0.0004	0.1859
18	aadA2	ARG	M3	2	0.0339	0.0002	0.0530

19	aadA	ARG	M3	2	0.0339	0.0002	0.0530
20	aadA17	ARG	M3	2	0.0339	0.0002	0.0530
21	tetA	ARG	M3	2	0.0339	0.0002	0.0530
31	g__norank_f__Methylophilaceae	Species	M5	2	0.0339	0.0002	0.1859
1	g__Flavobacterium	Species	M1	1	0.0170	0.0000	0.2616
3	g__Rhodoluna	Species	M8	1	0.0170	0.0000	0.0226
7	g__Exiguobacterium	Species	M1	1	0.0170	0.0000	0.2616
22	g__Sphingorhabdus	Species	M2	1	0.0170	0.0000	0.2354
24	g__Clostridium_sensu_stricto_13	Species	M2	1	0.0170	0.0000	0.2354
39	g__norank_f__Rhizobiales_Incertae_Sedis	Species	M1	1	0.0170	0.0000	0.2616
41	intI1	MGE	M7	1	0.0170	0.0000	0.1541
42	intl2	MGE	M7	1	0.0170	0.0000	0.1541
48	g__Dechloromonas	Species	M4	1	0.0170	0.0000	0.1780
51	g__Polynucleobacter	Species	M2	1	0.0170	0.0000	0.2354
52	g__Arenimonas	Species	M8	1	0.0170	0.0000	0.0226
54	qacH_351	ARG	M6	1	0.0170	0.0000	0.2242
55	EreA	ARG	M6	1	0.0170	0.0000	0.2242
56	trb-C	MGE	M6	1	0.0170	0.0000	0.2242
57	g__Novosphingobium	Species	M2	1	0.0170	0.0000	0.2354
